# Proteomic Analysis of Potential Targets for Non-Response to Infliximab in Patients With Ulcerative Colitis

**DOI:** 10.3389/fphar.2022.905133

**Published:** 2022-06-13

**Authors:** Lu Liu, Dan Pu, Dandan Wang, Muhan Zhang, Chuan Zhou, Zhe Zhang, Baisui Feng

**Affiliations:** ^1^ Department of Gastroenterology, The Second Affiliated Hospital of Zhengzhou University, Zhengzhou, China; ^2^ Neonatal Intensive Care Unit, The Second Affiliated Hospital of Zhengzhou University, Zhengzhou, China

**Keywords:** proteomics, ulcerative colitis, infliximab, drug targets, biomarkers

## Abstract

**Background:** Infliximab (IFX) is a potent therapeutic agent used for the treatment of conventional refractory ulcerative colitis (UC). However, the high non-response rate of IFX brings difficulties to clinical applications. In the context of proteomics research, our study of differentially expressed proteins (DEPs) is essential for non-response to IFX in UC patients and provides powerful insights into underlying drug resistance mechanisms.

**Methods:** A total of 12 UC patients were divided into responders to IFX (UCinfG), non-responders to IFX (UCinfL), severe UC (UCsevere) without an IFX treatment history, and mild UC (UCmild) without an IFX treatment history. Subsequently, DEPs were identified from intestinal biopsy tissue between responders and non-responders to IFX by a label-free proteomic quantitative approach, and the general principle of functional protein screening was followed to deduce the potential drug targets and predictors for non-response to IFX in UC patients. Meanwhile, these targets excluded DEPs caused by the severity of inflammation for the first time. The differential expressions of candidate protein targets were validated at the gene sequence level using GEO2R analysis of the GEO database and qRT-PCR in some independent cohorts.

**Results:** A total of 257 DEPs were screened out by mass spectrometry between UCinfG and UCinfL groups, excluding 22 DEPs caused by the severity of inflammation, and compared and verified at the gene sequence level in the Gene Expression Omnibus (GEO) database. Finally, five DEPs, including ACTBL2 (Q562R1), MBL2 (P11226), BPI (P17213), EIF3D (O15371), and CR1 (P17927), were identified as novel drug targets and predictive biomarkers for non-response to IFX. The drug targets were confirmed in the GEO database of the microarray results from three independent cohorts of 70 human intestinal biopsies and validated in qPCR data from 17 colonic mucosal biopsies. Among them, CR1 might affect the activation of the lectin pathway *via* complement-coated bacteria to play an opsonizing role in inflammation-related pathways closely associated with non-responders to IFX.

**Conclusion:** This is the first report of proteomics analysis for the identification of novel drug targets based on intestinal biopsy tissue, which is significant for hypotheses for mechanistic investigation that are responsible for non-response to IFX and the development of clinical new pharmaceutical drugs.

## 1 Introduction

Both Crohn’s disease (CD) and ulcerative colitis (UC) belong to inflammatory bowel disease (IBD), which is prevalent in the industrialized world composed of Western countries (Kaplan, 2015). At the turn of the 21st century, IBD has gradually become a global disease due to its rising prevalence in newly industrialized countries/regions, such as Asia and Latin America (Ng et al., 2017; Kaplan and Windsor, 2021).

The key feature of ulcerative colitis is diffuse mucosal inflammation of varying degrees that extends proximally from the rectum (Xavier and Podolsky, 2007). The etiology of ulcerative colitis remains incompletely explained. Current knowledge supports that UC is caused by an aberrant immune response of genetically predisposed individuals exposed to the environment in response to symbiotic microorganisms living in the gastrointestinal tract, collectively termed the gut microbiota (Khor et al., 2011; Jostins et al., 2012; Liu et al., 2015).

Monoclonal antibodies, targeting TNF-α, have been utilized to treat patients with refractory IBD who have experienced failed conventional therapies (Rutgeerts et al., 2005; Ford et al., 2011). Anti-tumor necrosis factor (anti-TNF) treatment, represented by infliximab (IFX), has been identified as a remission-inducing and maintaining therapy that is efficacious in 30%–50% of patients. IFX can not only improve intestinal mucosal healing and relieve symptoms but also reduce the hospitalization rate and enhance the overall quality of life of patients with IBD (Ben-Horin et al., 2014; Schmitt et al., 2019). Nevertheless, a subset of patients (about 20%) show non-response to IFX therapy, and a comparable proportion of patients may show decreasing response every year (Wong and Cross, 2017).

The high non-response rate of IFX therapy has inspired modern medical research methods at different levels (epidemiology, clinic, basic research, etc,.) to explore the variables used to elucidate mechanisms and predict individual patient’s responses to IFX therapy (Ding et al., 2016). So far, several published studies have collected data from IBD patients’ intestinal or blood samples before administering anti-TNF-α treatment. Several signature patterns of non-response patients were determined, through comprehensive comparison of genetics, gene expression and microorganisms, providing molecular mechanisms and predictive biomarkers of resistance to anti-TNF-α agents (Arijs et al., 2009a; Arijs et al., 2009b; Toedter et al., 2011; Mesko et al., 2013; Telesco et al., 2018; Aden et al., 2019; Pavlidis et al., 2019; Liu et al., 2020). However, it remains challenging to anticipate the response of IFX treatment in clinical application and to provide a new treatment for refractory IBD patients with IFX non-response (Bernstein et al., 2016; Ding et al., 2016).

In view of the clinical need to find the next targets for patients with IFX non-response and the research on the mechanism of IFX non-response, we comprehensively analyzed the proteins expressed highly in the intestinal mucosa of UC patients with non-response to IFX. As we all know, the biological and functional performance of cells is mainly governed by proteins, which directly participate in almost all physiological processes (Gazouli et al., 2013). Proteomics technology may be regarded to reflect some signatures of a disease, which is caused by the result of the interactions between the genotypic milieu and environmental factors (Viennois et al., 2015). The nature of the proteome makes proteomics research particularly useful for identifying potential drug targets and discovering novel biomarkers. Thus, we used proteomics technology to screen potential targets and biomarkers for UC patients with IFX non-response.

## 2 Materials and Methods

### 2.1 Study Population and Design

#### 2.1.1 Cohort A

Twelve patients with active ulcerative colitis were studied in cohort A (Table 1[A]), half of whom were refractory to corticosteroids and/or immunosuppression and treated with infliximab and were divided into responders to infliximab (UCinfG) and non-responders to infliximab (UCinfL). The other six enrolled UC patients without a history of infliximab treatment were divided into two groups: the severe UC group (UCsevere) and the mild UC group (UCmild).

**TABLE 1:** Basic clinical characteristics of the ulcerative colitis patients from cohorts A and B.

(A) Cohort A						
Group (n)	UCinfG (*n* = 3)	UCinfL (*n* = 3)	UCsevere (*n* = 3)	UCmild (*n* = 3)	*p* value	*p* value*
Male/female	2/1	2/1	2/1	2/1	—	—
Age	26.0 (6.2)	33.3 (15.6)	42.7 (17.2)	24.3 (7.1)	0.329	0.376
Disease duration	4.3 (2.5)	3.5 (2.6)	0.7 (0.5)	1.2 (0.8)	0.119	0.173
*Disease evolution* *^a^*						
Pre. T&W					0.045	—
Mild	0 (0.0%)	1 (33.3%)	0 (0.0%)	3 (100.0%)	—	—
Moderate	0 (0.0%)	1 (33.3%)	0 (0.0%)	0 (0.0%)	—	—
Severe	3 (100.0%)	1 (33.3%)	3 (100.0%)	0 (0.0%)	—	—
Post. T&W					0.02	—
Mild	1 (33.3%)	0 (0.0%)	0 (0.0%)	3 (100.0%)	—	—
Moderate	2 (66.7%)	1 (33.3%)	0 (0.0%)	0 (0.0%)	—	—
Severe	0 (0.0%)	2 (66.7%)	3 (100.0%)	0 (0.0%)	—	—
Pre. MMDAI	11.0 (11.0–11.0)	9.0 (8.5–10.0)	12.0 (11.5–12.0)	6.0 (5.5–6.0)	<0.001	0.024
Post. MMDAI	4.0 (3.0–6.0)	11.0 (10.5–11.5)	6.0 (6.0–8.0)	5.0 (4.5–5.0)	0.014	0.047
Pre. ESR	33.5 (26.2–40.8)	22.0 (14.5–71.0)	34.0 (25.5–47.0)	3.0 (2.5–3.5)	0.478	0.111
Post. ESR	18.0 (13.0–20.5)	29.0 (27.5–74.5)	34.0 (24.0–35.5)	3.0 (2.5–3.5)	0.168	0.041
Pre. CRP	19.3 (10.2–28.5)	15.4 (8.1–111.7)	19.3 (12.1–49.6)	0.8 (0.8–1.2)	0.602	0.286
Post. CRP	1.4 (1.1–6.0)	11.6 (9.5–76.4)	13.9 (7.8–26.8)	0.8 (0.8–1.0)	0.398	0.063
Pathology					0.007	—
Mild	3 (100.0%)	0 (0.0%)	0 (0.0%)	3 (100.0%)	—	—
Extensive	0 (0.0%)	3 (100.0%)	3 (100.0%)	0 (0.0%)	—	—
(B) Cohort B						
Group (n)	UCinfG (*n* = 7)	UCinfL (*n* = 10)	—	—	*p* value	*p* value*
Male/female	7/0	5/5	—	—	—	—
Age	33.3 (12.5)	48.8 (13.5)	—	—	0.03	0.05
Disease duration	4.7 (3.0)	5.0 (2.6)	—	—	0.827	0.326
*Disease evolution* *^a^*
Pre. T&W					0.385	—
Mild	0 (0.0%)	0 (0.0%)	—	—	—	—
Moderate	1 (14.3%)	4 (40.0%)	—	—	—	—
Severe	6 (85.7%)	6 (60.0%)	—	—	—	—
Post. T&W					<0.001	—
Mild	7 (100.0%)	0 (0.0%)	—	—	—	—
Moderate	0 (0.0%)	2 (20.0%)	—	—	—	—
Severe	0 (0.0%)	8 (80.0%)	—	—	—	—
Pre. MMDAI	10.0 (9.5–10.0)	10.5 (8.2–11.0)	—	—	0.987	0.422
Post. MMDAI	4.0 (3.0–4.0)	10.0 (8.2–10.0)	—	—	<0.001	<0.001
Pre. ESR	43.0 (14.5–48.0)	52.0 (16.8–79.0)	—	—	0.284	0.304
Post. ESR	2.0 (2.0–2.0)	40.0 (15.8–46.5)	—	—	<0.001	<0.001
Pre. CRP	36.2 (7.8–37.7)	24.8 (4.7–57.9)	—	—	0.536	0.377
Post. CRP	0.8 (0.5–0.9)	8.6 (3.6–27.4)	—	—	0.066	0.003
Pathology					<0.001	—
Mild	7 (100.0%)	0 (0.0%)	—	—	—	—
Extensive	0 (0.0%)	10 (100.0%)	—	—	—	—

a (Post-treatment of inflfliximab at least 14 weeks) Continuous variables were described as Mean (SD) and Median (IQR). Categorical variables were described as N (%). *p* value*: If it is a continuous variable, the Kruskal Wallis rank sum test should be performed; if the count variable has a theoretical number <10, the Fisher’s exact probability test must be applied to calculate it. SD, standard deviation; IQR, interquartile range; T&W, Truelove and Witts’ severity index; MMDAI, modified mayo disease activity index.

#### 2.1.2 Cohort B

For the current study, 17 post-infliximab treatment intestinal biopsies from 15 UC patients were obtained (three colonic mucosal biopsies were obtained during different periods of colonoscopy in the same patient after the fourth infliximab treatment), who received a loading dose of infliximab (5 or 10 mg/kg) for refractory ulcerative colitis. In cohort B, seven biopsy samples from five IFX responders were divided into the UCinfG group and the biopsy samples from the remaining IFX non-responders were divided into UCinfL group (Table 1[B]). Patients with moderate-to-severe active UC who were refractory to conventional therapy with corticosteroids were eligible to recieve IFX and had colonic biopsies obtained during endoscopy performed at least 14 weeks after their first IFX treatment.

A total of twenty-seven patients were hospitalized in the Gastroenterology Department of the Second Affiliated Hospital of Zhengzhou University from September 2019 to March 2021 and met the second European evidence-based consensus diagnostic criteria for the diagnosis and treatment of UC. Demographic, clinical, and colonoscopy data were obtained from each subject’s medical records. Each patient had the following characteristics recorded: age at diagnosis, gender, disease duration, previous and post-treatment levels of C-reactive protein (CRP) and erythrocyte sedimentation rate (ESR), as well as endoscopic pathological findings at inclusion.

For the proteomic analysis studies, response to therapy was defined as clinical remission and endoscopic mucosal healing and assessed for IFX after W4–6. Non-response was considered as lack of improvement or worsening of clinical or endoscopic appearance or disease symptoms.

Clinical remission was defined by the attending physician as an improvement in clinical and/or endoscopic symptoms associated with IBD, and comprehensively evaluates disease activity at least 14 weeks after the initiation of infliximab treatment. Disease activity is determined by the Modified Mayo Disease Activity Index (MMDAI) scoring system (Scherl et al., 2009), including gross disease extent, Mayo score at biopsy site, and endoscopic Mayo score. Notably, endoscopic mayo score and mayo score at the biopsy site were separated since they were not usually the same (i.e., the mayo score at the biopsy site was lower than the mayo score in the worst section of colitis). The MMDAI, which is the most widely used scoring system in clinical research, evaluates defecation frequency, rectal bleeding, endoscopic Mayo score, and global assessment by doctors on a scale of 0–3 with a maximum total score of 12. It is stipulated that the total score <2 is the remission period, 3–5 is a mild activity period, 6–10 is a moderate activity period, and 11–12 is a severe activity period. The global assessment by doctors also refers to Truelove and Witts’ criteria (T&W) (Croft et al., 2022), including defecation times, hematochezial state, body temperature, pulse, and biochemical data (ESR, hemoglobin, CRP). The UCsevere group consisted of patients who had moderate-to-severe active UC with a Mayo score of 6–12 points and an endoscopic score of ≥2 points, despite concomitant use of corticosteroids only or in combination with azathioprine and 5-aminosalicylate drugs. The remaining patients who had mild endoscopic and clinical scores were assigned to the UCmild group.

Endoscopic mucosal healing was based on the endoscopic pathological score. All biopsies have been examined by a gastroenterologist who specializes in inflammatory bowel disease. The histologic score was determined by the most severely afflicted tissues of the available biopsies in patients. The score was based on eight different parameters: the number of intraepithelial neutrophils per high power field, number of crypt abscesses, number of neutrophils within the lamina propria per high power field, lymphoplasmacytic inflammatory infiltrate within the lamina propria, number of ulcers, number of branched crypts, number of both short and spaced crypts. The lymphoplasmacytic inflammatory infiltration was the sole categorical parameter measured, and it was classified as normal, mild, and extensive. After comprehensive evaluation, including clinical symptoms, laboratory examinations, and endoscopic pathological manifestations, the severity of the disease is divided into mild, moderate, and severe.

### 2.2 Sample Preparation of Quantitative Mass Spectrometry

Enteroscope biopsy samples were removed from−80°C. Adequate tissue samples were weighed and placed in a mortar that had been pre-cooled using liquid nitrogen. After adding liquid nitrogen, the samples were fully ground to powder. Each group’s samples were treated with 4 times the volume of 10% TCA/acetone powder and stood at−20°C for 4 h. The mixture was centrifuged at 4500 g at 4°C for 5 min, and the supernatant was thrown away. It was necessary to wash the precipitate three times with pre-cooled acetone before it could be dried. Finally, the protein concentration was determined using a BCA kit after redissolving the precipitate with 8 M urea (1% protease inhibitor). Enzymatic hydrolysis was performed on an equal amount of protein from each sample. The volume was adjusted to the same as the lysate, and then the protein solution was reduced for 30 min at 56°C with 5 mM dithiothreitol and then alkylated for 15 min at room temperature in darkness with 11 mM iodoacetamide. The alkylated sample was transferred to an ultrafiltration tube, centrifuged at 12000g for 20 min at room temperature, replaced with 8 M urea for 3 times, and then replaced urea with a replacement buffer for 3 times. Trypsin was introduced at a 1:50 trypsin-to-protein mass ratio and enzymolysis was carried out overnight. The peptide was recovered by centrifugation at 12000 g for 10 min at room temperature. Then the peptide was recovered once with ultrapure water, and the peptide solution was combined twice.

### 2.3 Analysis by Liquid Chromatography-Mass Spectrometry

The EASY-nLC 1,200 ultra-high-performance liquid system was used to separate the peptides after they had been dissolved in liquid chromatography mobile phase A. An aqueous solution of 0.1% formic acid and 0.2% acetonitrile was used as the mobile phase A; the acetonitrile was used as the mobile phase B. At 500 nL/min, the liquid phase gradient was set at 0–68 min, 5% 22% B; 68% 34% B; 34% 80% B; and 80%–90 min, 80% B; the flow rate was kept constant. Once the peptides had been separated using ultra-high performance liquid chromatography, they were introduced into a capillary ion source for ionization before being injected into an Orbitrap exploration ™ 480 mass spectrometry system for further analysis. For the detection and analysis of peptide precursor ions and secondary fragments, a 1.6 kV ion source voltage and a high-resolution TOF technique were utilized. It was customary to set the screening range of the secondary mass spectrum between 100 and 1700. In this mode, parallel accumulation serial fragmentation (PASEF) was employed for data gathering. Following the collection of a first-level mass spectrum, there were 10 acquisitions in PASEF mode in order to obtain the second-level spectra with precursor ion charges ranging from 0 to 5, and the dynamic excluding time for scanning in tandem mass spectra was adjusted to 20 s so that the precursor ion was not repeatedly scanned.

### 2.4 Bioinformatics Analysis

The datasets generated for this study are available via ProteomeXchange with identifier PXD030121 in the PRIDE. The UniProt-GOA database (http://www.ebi.ac.uk/GOA/) was used to create the Gene Ontology (GO) annotation proteome. In the first step, identified protein IDs were transformed into UniProt IDs and then protein IDs were mapped to GO IDs. If some of the proteins identified were not annotated by the UniProt-GOA database, the InterProScan application would be applied to annotate the protein’s GO functionalities depending on the protein sequence alignment approach. After that, proteins were categorized using GO annotation into three categories: biological process, cellular component, and molecular function.

Based on the information in the Kyoto Encyclopedia of Genes and Genomes (KEGG) database, the protein pathway has been annotated. Firstly, the KEGG online system tool KAAS was applied to annotate a protein’s KEGG database definition. With the use of the KEGG online system’s KEGG mapper tool, the results of the annotation were subsequently mapped onto the KEGG pathway database.

Enrichment of pathway analysis: to identify enriched pathways, we used the KEGG database and a two-tailed Fisher’s exact test to examine if the DEPs were significantly more abundant than the other proteins that had been identified. A significant route was one with a corrected *p* value < 0.05. According to the KEGG website, these pathways are categorized into hierarchical categories.

Further hierarchical clustering was dependent on DEP’s functional categorization for further refinement (such as the KEGG pathway). Following the enrichment process, we collected all of the categories that were obtained as a result of the process, as well as their *p* values, and then screened for categories that were enriched in at least one of the clusters with *p* values < 0.05. The function x =−log10 was used to modify this filtered *p* value matrix (*p* value). Finally, each functional category’s x values were z-transformed. Afterwards, using one-way hierarchical clustering, the z scores were grouped together in Genesis (Euclidean distance, average linkage clustering). A heat map created with the “heatmap.2″ function from the “gplots” R package was used to display cluster membership.

For protein-protein interactions, a search was performed against the STRING database version 11.0 for every DEPs database accession and sequence. Only protein-protein interactions from the searched data set were chosen, hence removing extraneous candidates. To measure interaction confidence, STRING offers a metric called “confidence score.” With a confidence score of 0.7 (high confidence), we retrieved all interactions. In R package “networkD3,” the interaction network from STRING was shown.

### 2.5 Studies of CEL Files From GEO

We downloaded CEL files from colon biopsy microarrays from the Gene Expression Omnibus (GEO) database (https://www.ncbi.nlm.nih.gov/geo/query/acc.cgi?acc=GSE73661, accession number GSE73661; https://www.ncbi.nlm.nih.gov/geo/query/acc.cgi?acc=GSE16879, accession number GSE16879; https://www.ncbi.nlm.nih.gov/geo/query/acc.cgi?acc=GSE12251, accession number GSE12251). The microarrays were analyzed using the previously established gene sets (Table 2). GEO2R is a statistical analysis software for differential analysis of expression profile chips based on the GEO database. LogFC, whose full name is log2 foldchange, represents the fold change. If the mean expression of the treatment group is 8; the mean expression of the control group is 2, then foldchange is 4. The log2 fold change is 2. Therefore, our default logFC > 1, means that the difference between the two groups is meaningful. There is a default minimum standard: the absolute value of logFC > 1, and the corrected *p* value (adj.P. Val) < 0.05 is considered significant. The absolute value of logFC, as compared with the control group, the gene change is not necessarily increased. There are also reduced ones. So logFC will have directionality. If it is negative, it means that it has low expression compared to the control group. If it is positive, it means it is a high expression. All differentially expressed proteins identified between the UCinfG group and the UCinfL group were compared and verified at the gene sequence level in the GEOs database.

**TABLE 2:** Colon biopsy discovery cohorts (in microarray); proteomics analysis were confirmed at the whole-genome gene expression level in UC patient cohorts.

Response^b^						
Microarray/cohort^a^	Genes in proteomics	IBD type	R	NR	GEO dataset	Associated publication
UC-A^c^	18	UC	8	15	GSE73661	Arijs et al. (2018)
UC-B^c^	14	UC	12	11	GSE12251	Arijs et al. (2009a)
UC-C^c^	16	UC	8	16	GSE16879	Arijs et al. (2009b)

a Details of the previously reported microarray data, grouped according to the cohort in which they were identified to be predictive of infliximab response.

b R/NR, number in each cohort.

c Proteomics were confirmed at the whole-genome gene expression level between cohort UC-A, UC-B, and UC-C. NR, non-responder; R, responder.

### 2.6 Quantitative Real-Time PCR

To validate the proteomics data, qPCR was performed for ACTBL2 (Beta-actin-like protein 2), MBL2 (Mannose-binding protein C), BPI (Bactericidal permeability-increasing protein), EIF3D (Eukaryotic translation initiation factor 3 subunit D), CR1 (Complement receptor 1), and GAPDH, which was used as the endogenous reference gene. cDNA was synthesized from 0.5 μg of total RNA extracted using Trizol (Invitrogen) from cohort B samples by reverse transcription (TAKARA), following the manufacturer’s protocol. The primers for target genes were designed using Primer Premier 5.0 software (Premier) and constructed by Sigma-Aldrich. The primers of five target genes are in Table 3 qPCR was carried out using the ABI STEPONE PLUS system and the SYBR Green assay (TAKARA). Data were analyzed with ABI STEPONE PLUS system software and visualized *via* the Mann-Whitney U-test for unpaired samples and the Wilcoxon signed-rank test for paired samples using GraphPad Prism 7 (GraphPad Software Inc., La Jolla, CA). The relative expressions of target mRNA levels were calculated as a ratio relative to the GAPDH reference mRNA.

**TABLE 3:** qPCR primers designed to amplify mRNA of candidate protein targets.

Gene	Forward	Reverse
*ACTBL2*	5′-CAT​GAT​AGG​GCG​TCC​TCG​AC-3′	5′-TGA​GCC​TCA​TCT​CCC​ACG​TA-3′
*MBL2*	5′-AAA​AAG​TCC​GGA​TGG​TGA​TAG​T-3′	5′-CCA​CTT​TTT​GAT​ACG​TGC​CAT​T-3′
*BPI*	5′-CTG​GAC​TAC​GCC​AGC​CAG​CAG​G-3′	5′-CTG​AAG​CAC​TAC​GTT​GTA​GAG​C-3′
*EIF3D*	5′-GGA​TAT​TGT​CGT​CCA​GAG​AGT​T-3′	5′-AAT​TGT​GGT​TGA​TGT​AGG​TTG​C-3′
*CR1*	5′-GGA​CTG​GTG​CTA​AGG​ACA​GG-3′	5′-GGA​TCC​GAA​CTG​GAT​GCC​TT-3′

### 2.7 Statistical Analysis

Excel (Microsoft) software was used to manage and analyze data. Data were analyzed using the statistical software package R-3.4.3 (https://www.R-project.org, The R Foundation), Empower-Stats (www.empowerstats.com), and GraphPad Prism 7. Differences between groups were analyzed using Student’s t-test, the Mann–Whitney U-test, and the Wilcoxon signed-rank test. Statistical significance was judged when the *p* value < 0.05 (two-sided). *p* value*: If it is a continuous variable, the Kruskal–Wallis rank sum test should be performed; if the count variable has a theoretical number <10, Fisher’s exact probability test must be applied to calculate it. We used Pearson’s correlation coefficient, principal component analysis (PCA), and relative standard deviation (RSD), three statistical analysis methods, to evaluate the repeatability of protein quantification.

## 3 Results

### 3.1 Overall Characteristics of Patients

**FIGURE 1:**
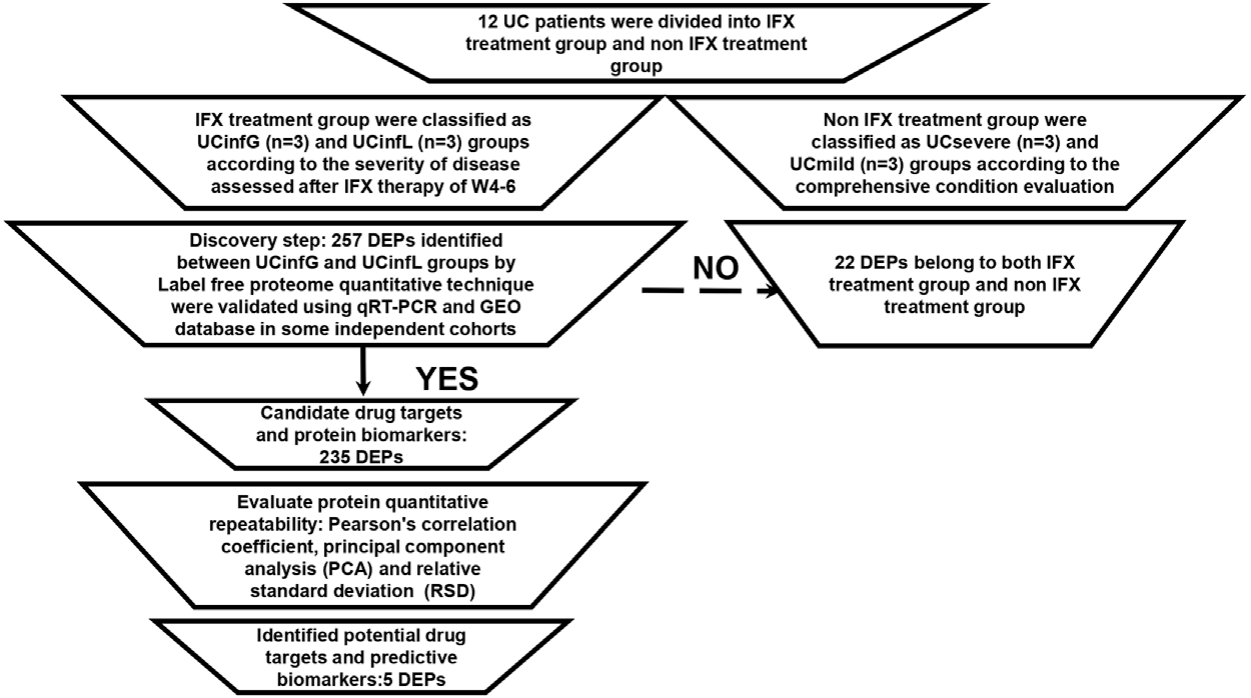
The workflow of proteomic analysis. Sample repeatability test: it was necessary to check whether the quantitative results of biological or technical replicate samples comply with statistical consistency. Here, we used Pearson’s Correlation Coefficient, principal component analysis (PCA), and relative standard deviation (RSD), three statistical analysis methods, to evaluate the repeatability of protein quantification.

Twenty nine human intestinal biopsies in cohorts A and B were included in this prospective and retrospective cohort study, and were divided into responders to IFX (UCinfG), non-responders to IFX (UCinfL), severe UC (UCsevere) without IFX treatment history and mild UC (UCmild) without IFX treatment history. Demographic and clinical characteristics from cohorts A and B are documented in Table 1. The Modified Mayo Disease Activity Index (MMDAI), Truelove and Witts’ criteria (T&W), C-reactive protein (CRP), and erythrocyte sedimentation rate (ESR) were significantly different among the groups. The workflow of histological analysis of these patients was shown in Figure 1.

### 3.2 DEPs Identified by LC-MS Analysis

**FIGURE 2:**
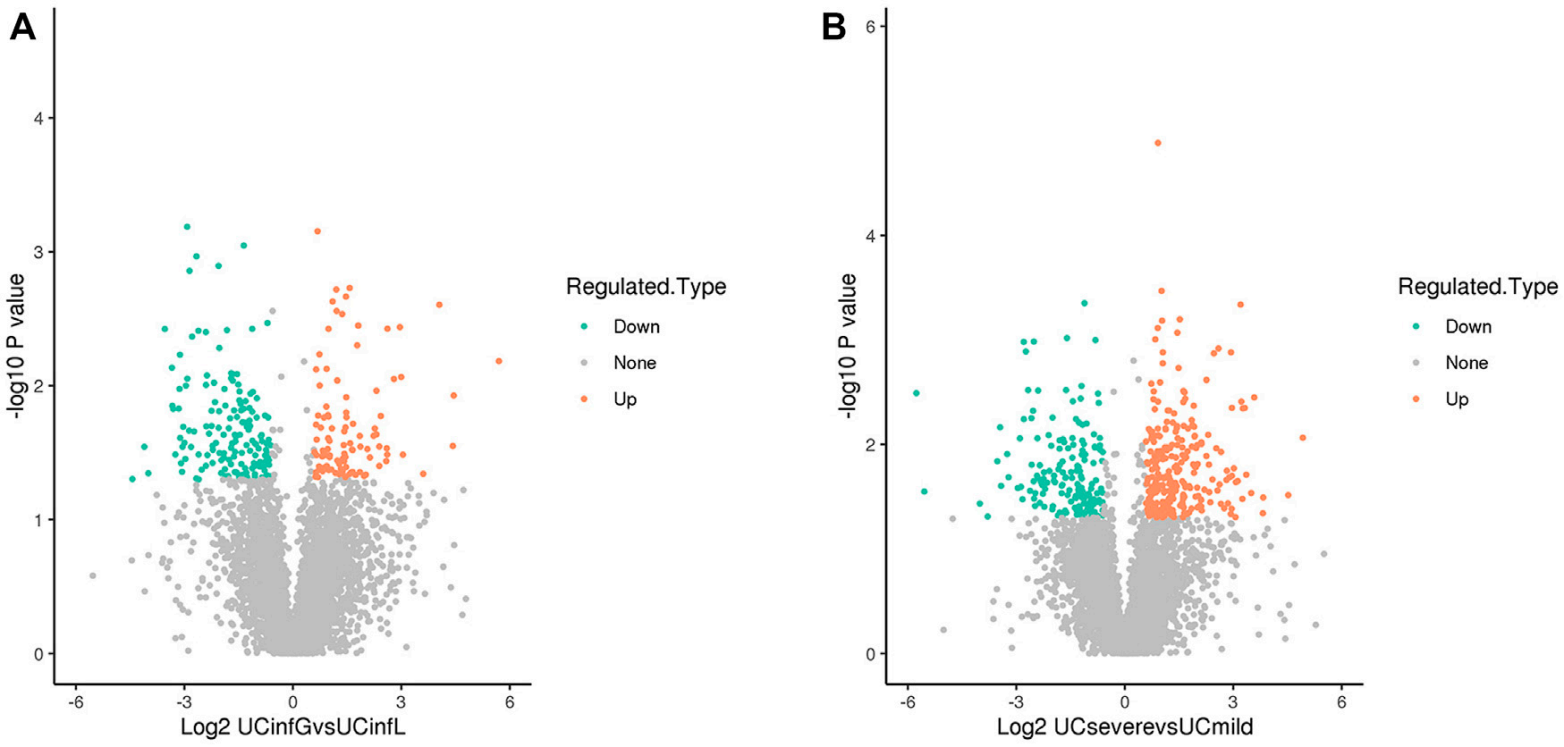
DEPs identified by LC-MS analysis. **(A)** A total of 257 DEPs were identified between UCinfG and UCinfL groups, including 104 upregulated proteins and 153 downregulated proteins. The relative expression level is represented by different colors. Green, low expression; gray, no difference; red, high expression. **(B)** A total of 437 DEPs were identified between UCsevere and UCmild groups, including 273 upregulated proteins and 164 downregulated proteins. The relative expression level is represented by different colors. Green, low expression; gray, no difference; red, high expression.

Through the LC-MS analysis of the colon tissue proteome among UCinfG, UCinfL, UCsevere, and UCmild groups composed of 12 subjects, a total of 1,014 DEPs were identified (Table 4). When *p* value ≤0.05, the change in differential expression level >1.5 was used as the change threshold for a significant upregulation, and <1/1.5 was used as the change threshold for a significant downregulation. A total of 257 DEPs were identified between UCinfG and UCinfL groups, including 104 upregulated proteins and 153 downregulated proteins (Figure 2A). Among them, the top five DEPs that were upregulated in the UCinfL group were Q562R1 (Beta-actin-like protein 2), P11226 (Mannose-binding protein C), P17213 (Bactericidal permeability-increasing protein), Q8WUJ3 (Cell migration-inducing and hyaluronan-binding protein), and P02679 (Fibrinogen gamma chain). The top five DEPs with downregulated expression in the UCinfL group were Q8WWA0 (Intelectin-1), Q14002 (Carcinoembryonic antigen-related cell adhesion molecule 7), Q9HBY8 (Serine/threonine-protein kinase Sgk2), Q6ZSS7 (Major facilitator superfamily domain-containing protein 6), and O00585 (C-C motif chemokine 21). Besides, a total of 437 DEPs were identified between UCsevere and UCmild groups, including 273 upregulated proteins and 164 downregulated proteins (Figure2B). All DEPs between UCinfG and UCinfL groups that have decreased or increased expression are listed in Supplementary Table S1. The datasets generated for this study are available *via* ProteomeXchange with identifier PXD030121 in the PRIDE.

**TABLE 4:** Differential protein screening.

Compared sample name	Upregulated	Downregulated
UCinfG/UCinfL	104	153
UCinfG/UCmild	73	58
UCinfL/UCsevere	67	122
UCsevere/UCmild	273	164

The sample pairs that need to be compared were picked out, and the ratio of the average of the quantitative values of all biological replicates of each protein in the comparison sample pair was took as the Fold Change (FC). For example, the multiple of protein difference between sample group A and sample group B was calculated. The calculation formula was as follows: where R represents the relative quantification of the protein, i represents the sample, and k represents the protein. FCA/B, k = Mean (Rik, i∈A)/Mean (Rik, i∈B) FCA/B, k = Mean (Rik, i∈A)/Mean (Rik, i∈B). To judge the significance of the difference, the relative quantitative value from every protein in the two samples was compared by T-test, and the associated *p* value was calculated as a significant index. By default, *p* value ≤ 0.05. In order to make the test data conform to the normal distribution of T-test requirements. Before the test, the corresponding quantitative value of the protein needs to undergo Log2 logarithmic conversion. The calculation formula is as follows: Pik = T. test (Log2 [Pik, i∈A], Log2 {Pik, i∈B}) Pik = T.test (Log2 [Pik, i∈A], Log2 {Pik, i∈B}). Through the above difference analysis, when *p* value ≤ 0.05, the variation in differential expression level >1.5 is employed as the change threshold for a considerable upregulation, and <1/1.5 is utilized as the change threshold for a significant downregulation.

### 3.3 Public IBD Gene Expression Data and Reported Protein Expression Data

**FIGURE 3:**
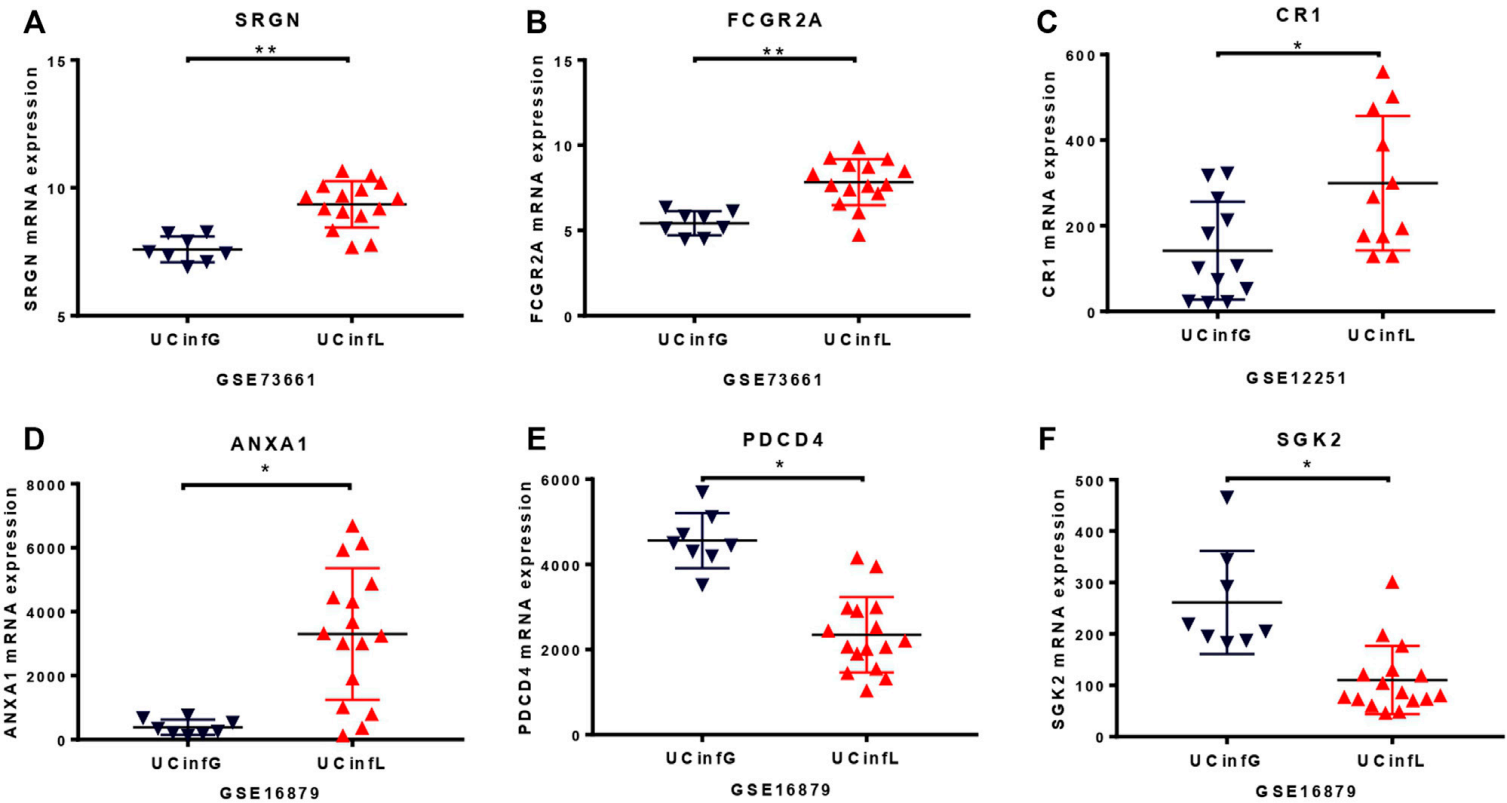
The RNA level of proteomic analysis in the GEO database. The whole-genome gene expression sequences by analyzing microarray data with GEO2R in the Gene Expression Omnibus database under accession number GSE73661 **(A,B)**, GSE12251 **(C)**, and GSE16879 **(D–F)** were similar to protein expression data from mass spectrometry that we described in our biopsy set. The target protein, complement receptor 1 (CR1/CD35), was compared and verified in microarray results from an independent cohort of IFX responders and non-responders in the GSE12251 database **(C)**. The small horizontal lines in the graphs indicate the average of each group. * adj.P. Val <0.05, ** adj.P. Val <0.01.

To further enhance the persuasiveness of our proteomic analysis, we used the data in the Gene Expression Omnibus (GEO) database at RNA level for further verification. We obtained colon biopsy gene expression data and patient infliximab responsiveness from published studies available in GEO and consisting of three cohorts of patients with UC (UC-A, UC-B, and UC-C) (Table 2). No significant differences in clinical parameters were observed in any of these cohorts at baseline. We studied the microarray results from 70 human colon biopsies from 69 patients. They were taken in three different cohorts after infliximab treatment and classified by response to therapy. They were downloaded from the GEO database (accession numbers GSE73661, GSE16879, and GSE12251). The whole-genome gene expression sequences by analyzing microarray data with GEO2R in biopsies of three cohorts of patients with UC after infliximab treatment were partially overlapped with protein expression data from mass spectrometry that was described in our biopsy set. SRGN (P10124), FCGR2A (P12318), ITGB2 (P05107), C4BPA (P04003), LCP2 (Q13094) and the other 13 DEPs have been identified as having a significant difference in the GSE73661 database (Figures 3A,B). What’s more, in the GSE12251 database, the target protein complement receptor 1 (CR1/CD35) was preliminary validated in the microarray results from an independent cohort of IFX responders and non-responders (Figure 3C). In the GSE16879 database, it is determined that SGK2 (Q9HBY8), PDCD4 (Q53EL6), ANXA1 (P04083), MTHFD2 (P13995), STOM (P27105), and another 11 DEPs have a substantial discrepancy (Figures 3D–F).

### 3.4 Validation of Target Proteins Using qPCR

**FIGURE 4:**
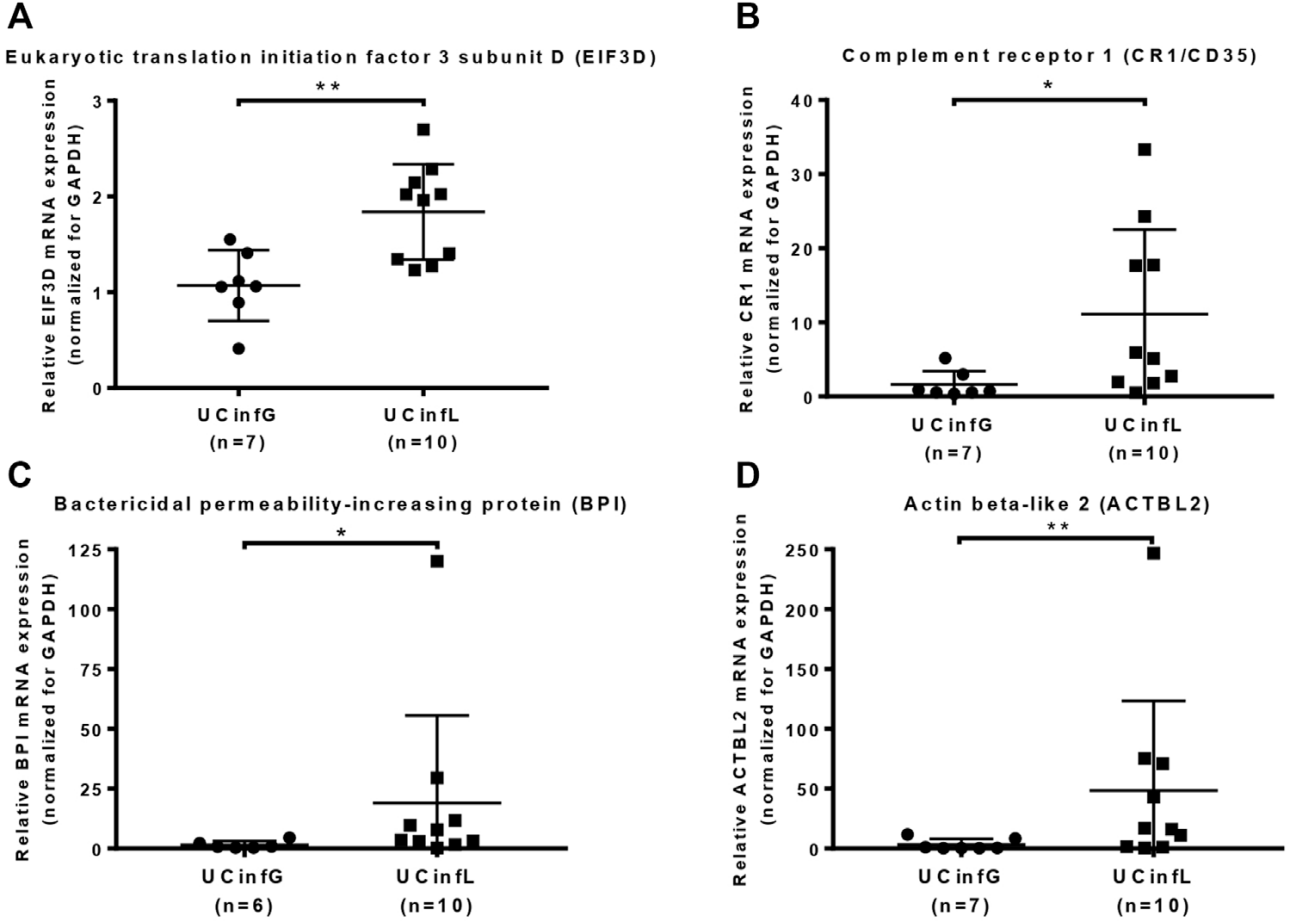
Validation of target proteins using qPCR. Quantitative RT-PCR was performed on the RNA samples from UC biopsy in cohort B. The target proteins Eukaryotic translation initiation factor 3 subunit D (EIF3D) **(A)**, complement receptor 1 (CR1/CD35) **(B)**, bactericidal permeability-increasing protein (BPI) **(C)**, and actin beta-like 2 (ACTBL2) **(D)** were validated at the gene sequence level. The small horizontal lines in the graphs indicate the average of each group. *p value < 0.05, **p value < 0.01.

Proteomic analysis of cohort A showed increased mRNA expression of the candidate target proteins in IFX non-responders when compared to IFX responders. To validate the proteomics data, quantitative RT-PCR was performed between IFX responders and non-responders of cohort B, and the target proteins Eukaryotic translation initiation factor 3 subunit D (EIF3D) (Figure 4A), Complement receptor 1 (CR1/CD35) (Figure 4B), Bactericidal permeability-increasing protein (BPI) (Figure 4C), and Actin beta-like 2 (ACTBL2) (Figure 4D) were confirmed to have a statistical difference in the expression of gene sequence level.

### 3.5 DEPs Functional Classifification

**FIGURE 5:**
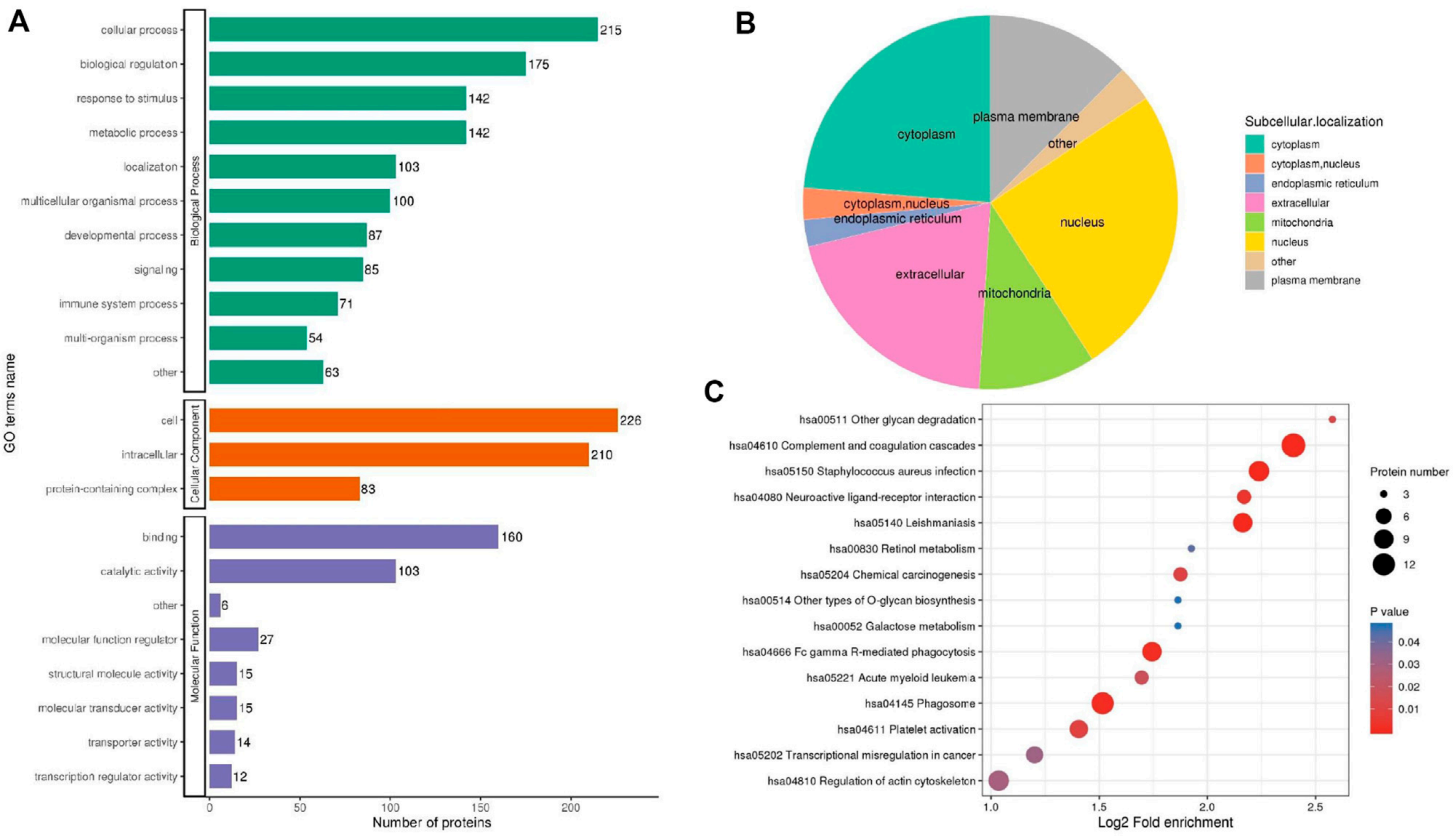
The Functional Classification Analysis of DEPs Between UCinfG and UCinfL groups. **(A)** The number of DEPs in the gene ontology classification of biological process (BP), cellular component (CC) and molecular function (MF). **(B)** In the subcellular localization classification, most differentially expressed proteins between UCinfG and UCinfL groups were located in the nucleus, cytoplasm, and extracellular. **(C)** The bubble diagram displayed the results of the first 15 classifications with the greatest enrichment. The vertical axis represents the function classification or route, and the horizontal axis represents the value of the ratio of the difference protein in the functional type compared to the change in the ratio of the identified protein after Log2 conversion. The circle’s color represents the *p* value of enrichment significance, and its size represents the number of differential proteins in the functional class or pathway.

GO annotation was divided into three categories: biological process (BP), molecular function (MF) and cellular component (CC), expounding on the biological characteristics of proteins from various perspectives. We compiled data on the distribution of DEPs between UCinfG and UCinfL groups in GO secondary annotations (Figure 5A). Notably, 71 of the 257 DEPs (28%) were engaged in the immune biological process, including S100-A14 (S100A14, Q9HCY8), leukosialin (SPN, P16150), interferon regulatory factor 7 (IRF7, Q92985), tyrosine-protein kinase HCK (HCK, P08631), C-C motif chemokine 21 (CCL21, O00585), intercellular adhesion molecule 3 (ICAM3, P32942), annexin A1 (ANXA1, P04083), mannose-binding protein C (MBL2, P11226), bactericidal permeability-increasing protein (BPI, P17213), and complement receptor type 1 (CR1, P17927) and the other 61 DEPs. For MF classification, binding (62%), catalytic activity (40%), and molecular function regulator (11%) were the principal functional categories of DEPs. For CC classification, cell (88%), intracellular (82%), and protein containing complex (32%) were the major components of DEPs. In the subcellular localization classification, the major differentially expressed proteins between the UCinfG and UCinfL groups were located in the nucleus, cytoplasm, and extracellular (Figure 5B).

### 3.6 DEPs Functional Enrichment Analysis

For the annotations of all detected proteins and the screening of DEPs, we performed enrichment analysis of the KEGG pathway for the DEPs between UCinfG and UCinfL groups (Figure 5C). The purpose was to find out whether there was a significant enrichment trend of DEPs in some functional types. For the enrichment test (Fisher’s exact test—which was employed in this case), the *p* value produced by the bubble chart shows the results of the functional classification and pathways of the first 15 classifications with significant enrichment of differential proteins (*p* < 0.05).

**FIGURE 6:**
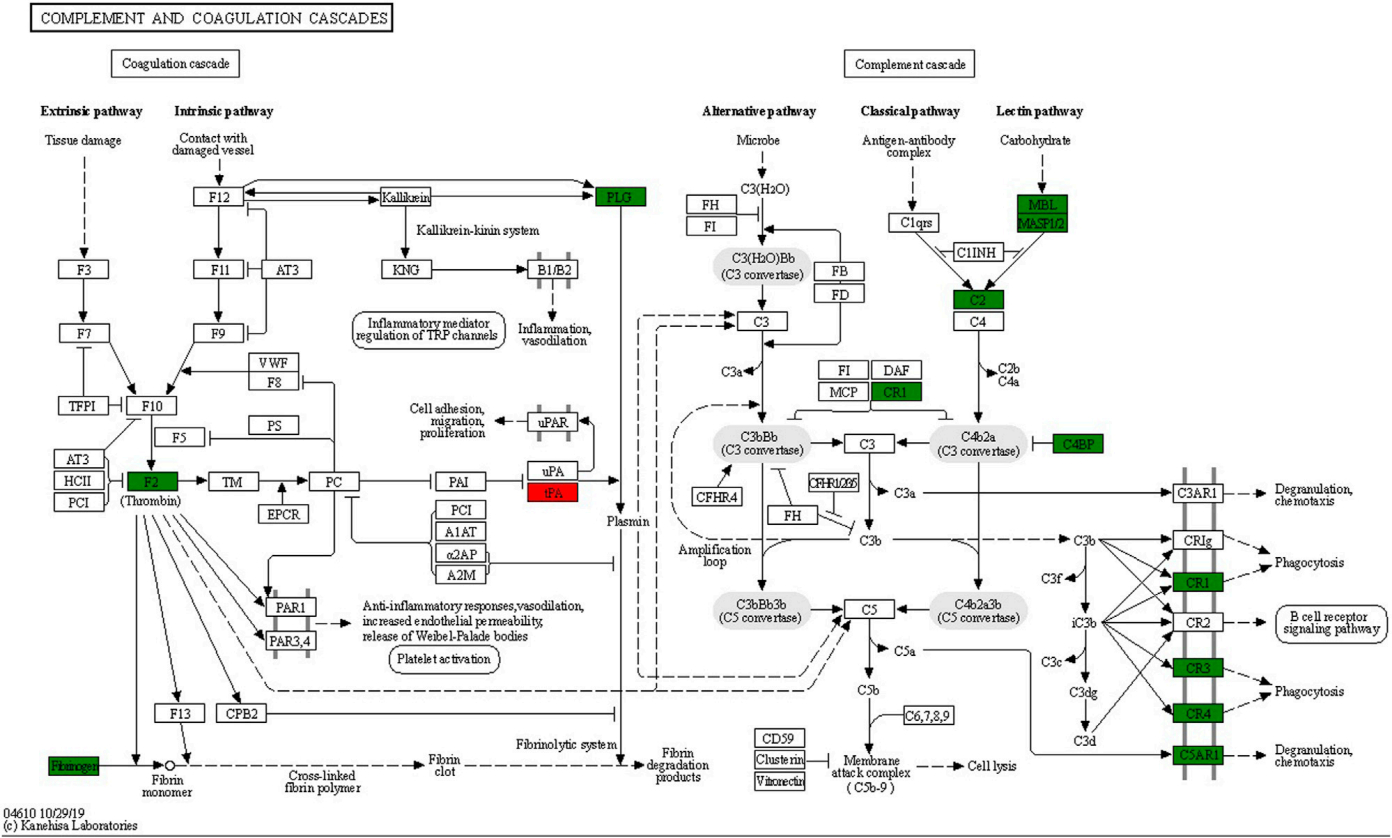
Lectin complement pathway and integrin αMβ2 signal diagram, including DEPs in complement and coagulation cascades enrichment pathway. Compared with the UCinfL group, upregulated DEPs in the UCinfG group were displayed in red boxes and downregulated DEPs in the UCinfG group were displayed in green boxes.

The top six enrichment pathways between UCinfG and UCinfL groups were complement and coagulation cascades, phagosome, *Staphylococcus aureus* infection, regulation of actin cytoskeleton, leishmaniasis, and the Fc gamma R-mediated phagocytosis pathway. Integrin alpha- M, integrin beta-2, and mannose-binding protein C were both involved in complement and coagulation cascades and phagosome pathway. In addition, DEPs enriched in the complement pathway (Figure 6) were the most related to the pathogenesis of IBD, mainly encoded by MBL2 (P11226) and CR1 (P17927). Mannose-binding protein C (MBL2/MBL) is a C-type or Ca2^+^-dependent lectin involved in recognition of microbial surfaces (Kawasaki et al., 1983), leading to complement activation, opsonization, and modulation of immunological responses (Gulla et al., 2009; Singh et al., 2016; Cedzyński et al., 2018). Complement receptor 1 (CR1/CD35) is a receptor related to immune complex clearance (Cedzyński et al., 2018). Additionally, it mediates the attachment and internalization of C4b/C3b binding ligands on neutrophils (Verschoor et al., 2017). During neutrophil activation, MBL was shown to behave as MBL-opsonized complexes that were recognized by CR1 (Ghiran et al., 2000). This may account for the occurrence of neutrophil activation in IFX non-responders’ inflamed intestinal tissues.

### 3.7 DEPs Cluster Analysis

**FIGURE 7:**
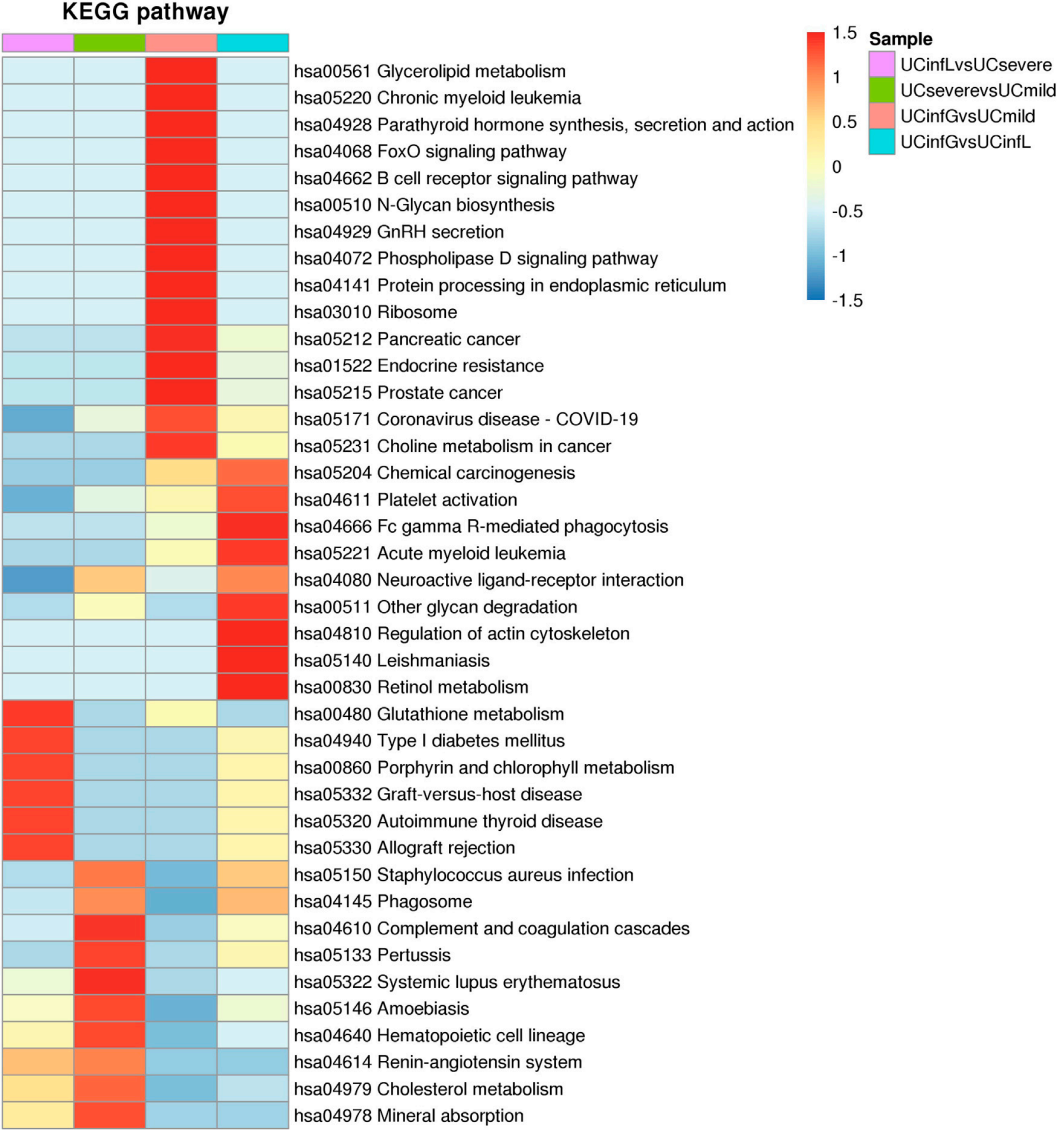
The cluster analysis of DEPs. The horizontal direction of the heat map represented the enrichment test findings of different groups, and the vertical direction was the description of the KEGG pathway related to differential expression enrichment. Different groups of DEPs of color blocks related to the functional description indicated the degree of enrichment. Red represented a strong degree of enrichment, and blue represented a weak degree of enrichment.

After these DEPs in different groups were enriched in the KEGG pathway, we performed cluster analysis (Figure 7) to find the correlation between the functions of DEPs among the comparison groups. The hierarchical clustering approach was utilized to classify the relevant functions into distinct categories based on the *p* value of Fisher’s exact test acquired from enrichment analysis and heatmap was drawn. Among them, the pathways significantly enriched between UCinfG and UCinfL groups, and also significantly enriched between UCsevere and UCmild groups, include neuroactive ligand-receptor interaction, platelet activation, and other glycan (including plasma alphac-L-fucosidase, beta-galactosidase, and tissue alpha-L-fucosidase) degradation pathways.

In addition, the pathways that were only significantly enriched between UCinfG and UCinfL groups were leishmaniasis, Fc gamma R-mediated phagocytosis, chemical carcinogenesis, acute myeloid leukemia, phospholipase D signaling pathway, regulation of actin cytoskeleton, and retinol metabolism pathway.

### 3.8 Protein–Protein Interaction Network Analysis

**FIGURE 8:**
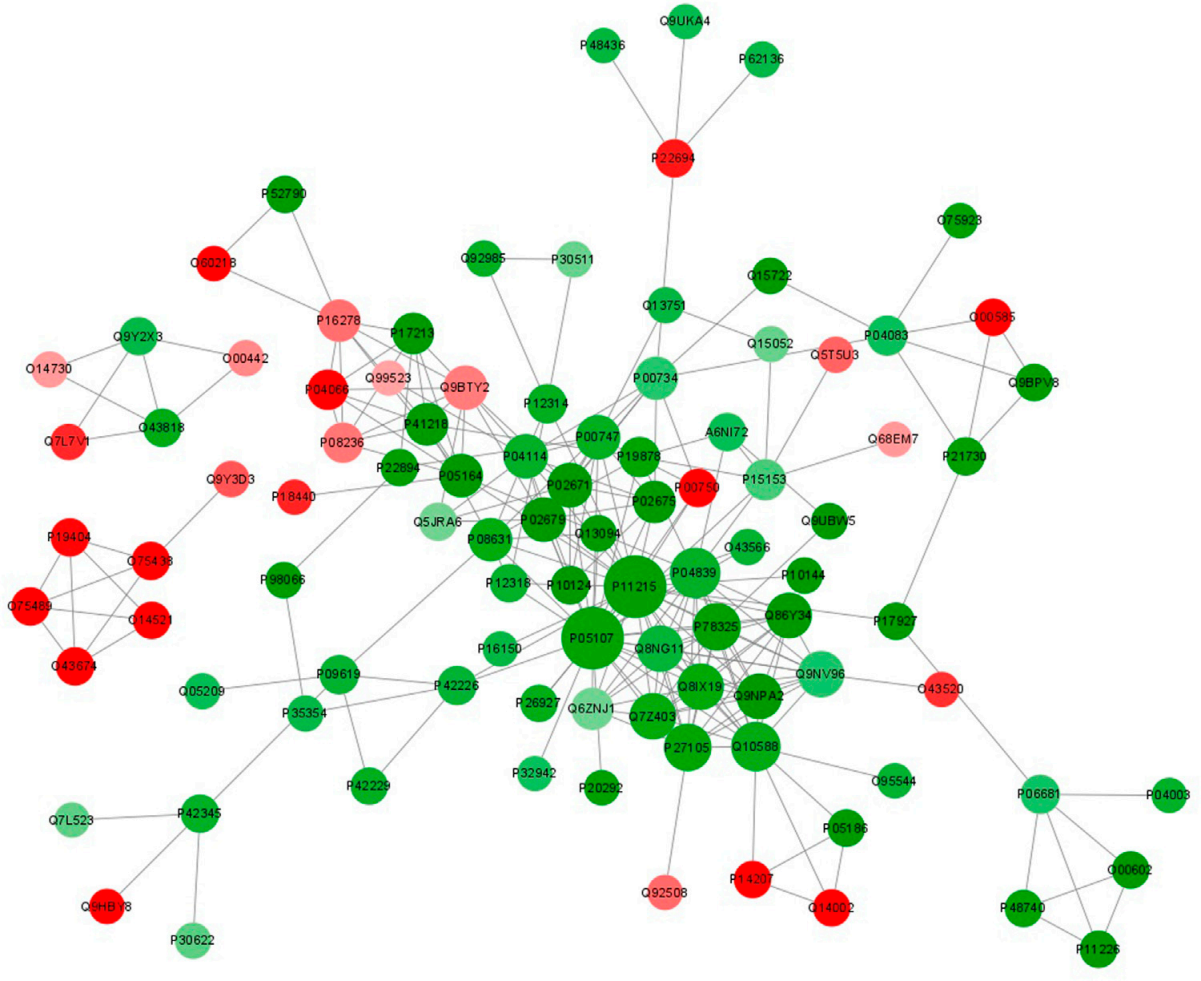
Protein-protein interaction network analysis of DEPs between UCinfG and UCinfL groups. The circles in the figure represent the DEPs, and different colors represent the DEPs (green is the downregulated protein and red is the upregulated protein). The size of the circle represents the number of differential proteins and their interacting proteins. The larger the circle, the more proteins interact with it, showing that the protein is more important in the network.

The function of proteins *in vivo* is determined by their interaction with other proteins. As a result, we constructed a protein-protein interaction network (PPI) for DEPs between UCinfG and UCinfL groups (Figure 8). We mapped the protein interaction network by screening the top 97 proteins with the closest interactions. ITGAM, ITGB2, CYBB, BST1, ADAM8, STOM, TMC6, TSPAN14, MCEMP1, TMEM30A were the top ten differential proteins with the closest interactions. Among them, integrin αMβ2 (CD11b/CD18, also known as Mac-1; complement receptor CR3) has the highest connectivity and interacts and connects with 28 proteins (Figure 6), which is necessary for the uptake of iC3b complement regulated granules by human dendritic cells (Lukácsi et al., 2017). In the endothelium, it is a membrane protein that plays a role in monocyte/neutrophil adhesion. The infiltration of monocytes and neutrophils into inflamed tissue may have a key role in the pathogenesis of IBD. In addition, during experimental colitis, CD11b might be important in regulating the number of distal colonic plasma cells (Abdelbaqi et al., 2006). It has been proven that anti-Mac-1 antibodies can reduce the clinical symptoms of experimental IBD in rats by partially blocking the infiltration of macrophages and granulocytes (Palmen et al., 1995)^.^ Therefore, DEPs enriched in the integrin αMβ2 signaling pathway might play an important role in IBD.

## 4 Discussion

A number of studies focusing on genetic markers, transcriptomics and proteomics have provided some evidence for the study of anti-TNF-α non-response (Bek et al., 2016; Gaujoux et al., 2019). However, so far, there is no mature clinical application to predict the occurrence of infliximab non-response or better alternative drugs after infliximab non-response. Although new biologics such as vedolizumab, are constantly used on patients, (Arijs et al., 2018), their onset time cannot reach the same level as infliximab according to the feedback from a large number of clinicians. Therefore, new therapeutic targets and drugs need to be continuously developed for the non-responders to infliximab.

To our knowledge, this is the first large-scale proteomic analysis using protein mass spectrometry to study the mechanism of drug non-response period of new biological agents represented by infliximab and to identify potential drug targets and predictive biomarkers. We identified five DEPs with the potential to become drug targets or biomarkers, which may help to provide new treatment options for IBD patients in the IFX non-response stage.

Among the 257 kinds of DEPs between UCinfG and UCinfL groups, 22 DEPs belong together between UCsevere and UCmild groups, including integrin alpha-M (ITGAM, P11215), C4b-binding protein alpha chain (C4BPA, P04003), PML-RARA-regulated adapter molecule 1 (PRAM1, Q96QH2), cell migration-inducing and hyaluronan-binding protein (CEMIP, Q8WUJ3), fibrinogen gamma chain (FGG, P02679), plasminogen (PLG, P00747), fibrinogen beta chain (FGB, P02675), fibrinogen alpha chain (FGA, P02671), early endosome antigen 1 (EEA1, Q15075), high affinity immunoglobulin gamma Fc receptor I (FCGR1A, P12314) and the other 12 DEPs. These proteins are mainly involved in complement and coagulation cascades, phagosomes, Fc gamma R-mediated phagocytosis pathway, etc,. The expression of these DEPs in both UCsevere and UCinfL was significantly increased, so they might be good potential targets for patients with severe UC, so as to exclude the specificity that they are closely related to the non-response of IFX.

Among the remaining 235 DEPs, we identified five new drug targets and biomarkers, following the general principles of functional protein screening: 1) selecting proteins with significantly different protein expression or modification levels, 2) prioritizing selection of proteins with relevant functional reports or potential relationship with the experimental system of this research, and 3) selecting some key proteins with significant differential expression in specific functions, pathways and components obtained in bioinformatics analysis.

Five novel drug targets and biomarkers for non-response of IFX included ACTBL2 (Q562R1), MBL2 (P11226), BPI (P17213), EIF3D (O15371), and CR1 (P17927). And they were significantly increased in the IFX non-response group compared with the IFX response group with a result *p* value ≤ 0.05 of the T-test of the relative quantitative value of the protein sample comparison and the differential expression change> 9.

Actin beta-like 2 (ACTBL2), which is a newly identified seventh isoform of actin, shares 92% structural similarity with ß-actin (Chang et al., 2006). In the original report of Bober et al. (2016) for fibroblast growth factor 1(FGF1), ACTBL2 and caspase-3 were identified as new binding molecules of FGF1 and affected its intracellular function. The growth factor FGF-1 involves in proinflammatory response in the spinal cord by inducing astrocytes to release ATP, which stimulates microglia activation and secreting TNF-α and IL-1β (Garré et al., 2016). ACTBL2 as a binding partner of gelsolin (GSN) (Mazur et al., 2016), has also been attributed to increasing cell proliferation in human colorectal adenocarcinoma and melanoma cells (Litwin et al., 2012). ACTBL2 may exacerbate intestinal inflammation in patients with UC by interfering with proteins that have the capacity to induce the proliferation or/and migration of inflammatory cells. The application suggests the potential of downregulating the expression of ACTBL2 protein in the treatment of IBD, especially in non-responders to IFX.

As a calcium-dependent lectin of the collecting family, mannose-binding lectin (MBL) forms complexes with mannose-binding lectin-associated serine proteases (MASP), which are involved in the activation of the complement system (Cedzyński et al., 2018). On the one hand, as a pattern-recognition molecule, MBL has broad specificity and contributes to the elimination of certain pathogens in the innate immune system, which is critical for infection prevention, particularly during infancy (Koch et al., 2001). In addition, MBL may cause an excessive and chronic inflammatory response to some stimuli, which adversely affects disease courses like IBD (Bąk-Romaniszyn et al., 2020). Thus, the role of high MBL activity seems to be dual, and it cannot be excluded that the composition of microbiota and other changes together form part of the multifactorial network conducive to the development of IBD (Milanese et al., 2007; Bąk-Romaniszyn et al., 2020). Inhibiting MBL might have a therapeutic effect on non-responders to IFX by inhibiting the innate immune response.

Bactericidal permeability-increasing protein (BPI) is a single-chain cationic 55 kDa protein that mainly exists on the surface of monocytes and gastrointestinal mucosal cells (Weiss et al., 1978; Dentener et al., 1996; Monajemi et al., 1996). Additionally, BPI is the most effective anti-microbial protein, especially against gram-negative bacteria (Weiss et al., 1978; Weiss et al., 1992), with lipopolysaccharide (LPS), and also forms the BPI-LPS complexes to compete with LPS-binding protein (LBP) for the binding of endotoxin, which limits endotoxin-triggered systemic inflammation *in vivo* (Weiss et al., 1978; Gazzano-Santoro et al., 1992). The concentrations of BPI protein increased in tissue samples from patients with IBD, and the concentrations of BPI was reported to be well related to histological inflammatory activity and endoscopic inflammatory score (Monajemi et al., 1996). The reasons may include two aspects. Auto-antibodies against BPI (p-ANCA) were described to be increased in association with the aggravation of IBD disease activity (Stoffel et al., 1996), and these auto-antibodies may impede the antibiotic activity of BPI (Schinke et al., 2004). In addition, Klein et al. (2005) discovered a link between polymorphism of BPI and Crohn’s disease. Amino acid exchange caused by genetic variation may result in functional alterations and, eventually, enhanced BPI molecule expression (Klein et al., 2005). Inhibition of BPI as a target might reduce the effect of BPI-LPS complexes on downstream proteins, thereby improving the condition of UC patients without a response to IFX.

It is commonly described that eIF3D is a subunit of the eIF3 complex, which is ubiquitously expressed in colon cells, participating in nearly each stage of transcription initiation (Hinnebusch, 2006; Yin et al., 2013; Yu et al., 2014). The over-expression of eIF3D is well-known to promote cell proliferation or/and migration of a variety of advanced gastrointestinal tumor entities, including gastric cancer (GC) (He et al., 2017), and gallbladder cancer (GBC) (Zhang et al., 2017). Previously published research shows that knockdown of eIF3D suppresses cell proliferation and colony formation via inducing cell-cycle arrest and apoptosis, involving colon cancer and malignant mesothelioma cells (Sudo et al., 2010; Yu et al., 2014). According to the results of our proteomic analysis, eIF3D may also play a role in the resistance to IFX.

Complement receptor 1 (CR1/CD35) is a large transmembrane glycoprotein, and is composed of several homologous motifs (Birmingham and Hebert, 2001), widely expressing in human erythrocyte (Ghiran et al., 2008). It is also present on monocytes, granulocytes, and B lymphocytes in humans (Ross et al., 1978; Birmingham and Hebert, 2001). CR1 can not only bind to C3b with high-affinity (Newman and Mikus, 1985), but also with lower affinity to iC3b, C4b, iC4b, C1q, and MBL (Cooper, 1969; Newman and Mikus, 1985; Ghiran et al., 2000). Due to the activation of classical, alternative, and lectin pathways, the complement-coated bacteria are connected to erythrocytes by playing an opsonizing role, hence recognized by the immune system and bound to specific receptors such as CR1 (Birmingham and Hebert, 2001). Since we mentioned earlier that increased MBL expression in IBD patients can affect the inflammatory response via the lectin pathway (Bąk-Romaniszyn et al., 2020), our results further support the therapeutical potential of targeting MBL–CR1 binding in UC patients.

In addition to some highly-expressed proteins in non-responders to IFX that may serve as new targets, we also explored some proteins that were highly expressed in patients with severe UC without the application of biological agents such as IFX, so as to provide direction for the research of new drugs for severe UC in the future.

Inflammation and coagulation are critical in the pathogenesis of several chronic inflammatory diseases. IBD is distinguished by two key features: a hypercoagulable state and a prothrombotic condition, both of which are associated with abnormalities in coagulation (Danese et al., 2007). Compared with mild to moderate active UC patients, we found coagulation factor V, coagulation factor XII, and plasma protease C1 inhibitor were upregulated proteins in severe active UC patients. Coagulation factor V, known as the key regulator of hemostasis, serves as a vital cofactor for the prothrombinase activity of factor Xa that leads to the activation of prothrombin to thrombin. Coagulation factor XII is a serum glycoprotein that aids in the initiation of blood coagulation, fibrinolysis, and the production of bradykinin and angiotensin. Plasma protein C1 inhibitor is an efficient inhibitor of FXIIa. It inhibits chymotrypsin and kallikrein, and may play a potentially critical role in regulating essential physiological pathways, including complement activation, blood coagulation, fibrinolysis, and the generation of kinins. Raised serum concentrations of plasma protein C1 inhibitor have been found in patients with active IBD (Potter et al., 1980).

PPARs are involved in the regulation of energy metabolism, inflammatory processes, and immune responses stimulated by natural ligands such as fatty acids, eicosanoids, and phospholipids (Green and Wahli, 1994; Desvergne and Wahli, 1999). Our results demonstrated that the differential proteins of PPAR signaling pathways, including phospholipid transfer protein, interstitial collagenase, glycerol kinase, apolipoprotein A-I, and platelet glycoprotein 4, were significantly enriched in UC patients with severe illness.

Platelet glycoprotein 4, as a coreceptor for the TLR4:TLR6 heterodimer, induces inflammation in monocytes/macrophages. Upon ligand binding, such as oxLDL or amyloid-beta 42, reacts with the heterodimer TLR4:TLR6, the complex is internalized and activates the inflammatory response, resulting in NF-κB-dependent production of CXCL1, CXCL2 and CCL9 cytokines, via MYD88 signaling pathway, and CCL5 cytokine, via the TICAM1 signaling pathway, as well as IL1B secretion, through the priming and activation of the NLRP3 inflammasome (Stewart et al., 2010). It is speculated that platelet glycoprotein 4 promotes the occurrence of IBD through these inflammatory cascades signaling pathways.

The merit of our study is that it applies a well-established diagnostic system to a heterogeneous disease (UC) in a varied patient cohort. We evaluated a group of patients who were varied in terms of clinical symptoms, laboratory tests, and endoscopic pathological appearance, then characterized and grouped by a single gastroenterologist and pathologist prior to protein molecular analysis.

Due to the limited number of existing colonic biopsies set, we have preliminarily identified 48 gene-level sequences identical to the DEPs recorded in the results of our protein mass spectrometry at the whole-genome gene expression level by using GEO2R analysis of the microarray results from 70 human colon biopsies of 69 patients, taken in three different cohorts after infliximab treatment and classified by response to therapy. And using qPCR analysis from 17 intestinal biopsies of 15 patients, we confirmed that the Eukaryotic translation initiation factor 3 subunit D, Complement receptor 1, Bactericidal permeability-increasing protein, and Actin beta-like 2 have a statistical difference in the expression of gene sequence level between responders and non-responders to IFX.

At the same time, our paper has special significance because it is the first report detected at the protein level on the identification of new drug targets and protein biomarkers, and we have also discovered some proteins different to the data in the GEO database which may specifically affect the response to infliximab in Chinese people. These genes will be verified in a larger number of completely standardized clinical cohorts by quantitative RT-PCR and western blot.

Lastly, our results do not yet explain how these differential immune milieus we observed between the response and non-response groups develop; this would require longitudinal studies tracking patients with early UC. Further study of the five quite novel proteins, including ACTBL2 (Q562R1), MBL2 (P11226), BPI (P17213), EIF3D (O15371) and CR1 (P17927), which are potential therapeutic targets and candidate biomarkers for IBD patients with IFX non-response, and pathways identified in the present study should allow a better understanding of the molecular mechanisms of infliximab action and mechanisms of resistance to anti-TNF therapy. These findings will lead to the development of dozens of novel therapeutic compounds for IFX non-response phase studies in patients with ulcerative colitis.

## Data Availability

The datasets generated for this study are available *via* ProteomeXchange with identifier PXD030121 in the PRIDE.
