# Supplementary material for: Proteomic Analysis of Potential Targets for Non-Response to Infliximab in Patients With Ulcerative Colitis
Source: Front Pharmacol. 2022 Jun 13;13:905133. doi: 10.3389/fphar.2022.905133 (PMC9234463; doi:10.3389/fphar.2022.905133)
Supplement: Supplementary file 1 [file Table1.DOCX]

| **UniProt KB** | **Protein description** | **Gene name** | **Ratio (UCinfG/UCinfL )** | **P-value (UCinfG/UCinfL )** |
| --- | --- | --- | --- | --- |
| Down-regulated proteins | | | | |
| Q562R1 | Beta-actin-like protein 2 OS=Homo sapiens OX=9606 GN=ACTBL2 PE=1 SV=2 | ACTBL2 | 0.0066 | 0.0003 |
| P11226 | Mannose-binding protein C OS=Homo sapiens OX=9606 GN=MBL2 PE=1 SV=2 | MBL2 | 0.0461 | 0.0498 |
| P17213 | Bactericidal permeability-increasing protein OS=Homo sapiens OX=9606 GN=BPI PE=1 SV=4 | BPI | 0.058 | 0.0286 |
| Q8WUJ3 | Cell migration-inducing and hyaluronan-binding protein OS=Homo sapiens OX=9606 GN=CEMIP PE=1 SV=2 | CEMIP | 0.0626 | 0.0451 |
| P02679 | Fibrinogen gamma chain OS=Homo sapiens OX=9606 GN=FGG PE=1 SV=3 | FGG | 0.0859 | 0.0038 |
| Q96QH2 | PML-RARA-regulated adapter molecule 1 OS=Homo sapiens OX=9606 GN=PRAM1 PE=1 SV=3 | PRAM1 | 0.0984 | 0.0074 |
| O15371 | Eukaryotic translation initiation factor 3 subunit D OS=Homo sapiens OX=9606 GN=EIF3D PE=1 SV=1 | EIF3D | 0.0992 | 0.0141 |
| P17927 | Complement receptor type 1 OS=Homo sapiens OX=9606 GN=CR1 PE=1 SV=3 | CR1 | 0.1008 | 0.0149 |
| Q8N386 | Leucine-rich repeat-containing protein 25 OS=Homo sapiens OX=9606 GN=LRRC25 PE=1 SV=2 | LRRC25 | 0.1051 | 0.0327 |
| Q9Y6D5 | Brefeldin A-inhibited guanine nucleotide-exchange protein 2 OS=Homo sapiens OX=9606 GN=ARFGEF2 PE=1 SV=3 | ARFGEF2 | 0.1121 | 0.0148 |
| Q8WXD5 | Gem-associated protein 6 OS=Homo sapiens OX=9606 GN=GEMIN6 PE=1 SV=1 | GEMIN6 | 0.1138 | 0.0106 |
| O00602 | Ficolin-1 OS=Homo sapiens OX=9606 GN=FCN1 PE=1 SV=2 | FCN1 | 0.1147 | 0.0059 |
| P10124 | Serglycin OS=Homo sapiens OX=9606 GN=SRGN PE=1 SV=3 | SRGN | 0.1152 | 0.0246 |
| Q86Y34 | Adhesion G protein-coupled receptor G3 OS=Homo sapiens OX=9606 GN=ADGRG3 PE=1 SV=1 | ADGRG3 | 0.1171 | 0.0379 |
| O75762 | Transient receptor potential cation channel subfamily A member 1 OS=Homo sapiens OX=9606 GN=TRPA1 PE=1 SV=3 | TRPA1 | 0.1195 | 0.0439 |
| O95749 | Geranylgeranyl pyrophosphate synthase OS=Homo sapiens OX=9606 GN=GGPS1 PE=1 SV=1 | GGPS1 | 0.1207 | 0.0286 |
| Q9UBW5 | Bridging integrator 2 OS=Homo sapiens OX=9606 GN=BIN2 PE=1 SV=3 | BIN2 | 0.1222 | 0.0203 |
| Q9NPA2 | Matrix metalloproteinase-25 OS=Homo sapiens OX=9606 GN=MMP25 PE=1 SV=1 | MMP25 | 0.125 | 0.0267 |
| Q9H939 | Proline-serine-threonine phosphatase-interacting protein 2 OS=Homo sapiens OX=9606 GN=PSTPIP2 PE=1 SV=4 | PSTPIP2 | 0.1273 | 0.0352 |
| Q15645 | Pachytene checkpoint protein 2 homolog OS=Homo sapiens OX=9606 GN=TRIP13 PE=1 SV=2 | TRIP13 | 0.1286 | 0.0100 |
| P02675 | Fibrinogen beta chain OS=Homo sapiens OX=9606 GN=FGB PE=1 SV=2 | FGB | 0.1318 | 0.0007 |
| Q13315 | Serine-protein kinase ATM OS=Homo sapiens OX=9606 GN=ATM PE=1 SV=4 | ATM | 0.132 | 0.0089 |
| Q15722 | Leukotriene B4 receptor 1 OS=Homo sapiens OX=9606 GN=LTB4R PE=1 SV=2 | LTB4R | 0.1368 | 0.0217 |
| P02671 | Fibrinogen alpha chain OS=Homo sapiens OX=9606 GN=FGA PE=1 SV=2 | FGA | 0.1378 | 0.0014 |
| P05164 | Myeloperoxidase OS=Homo sapiens OX=9606 GN=MPO PE=1 SV=1 | MPO | 0.1419 | 0.0286 |
| P41218 | Myeloid cell nuclear differentiation antigen OS=Homo sapiens OX=9606 GN=MNDA PE=1 SV=1 | MNDA | 0.1448 | 0.0043 |
| P10144 | Granzyme B OS=Homo sapiens OX=9606 GN=GZMB PE=1 SV=2 | GZMB | 0.1504 | 0.0219 |
| Q13094 | Lymphocyte cytosolic protein 2 OS=Homo sapiens OX=9606 GN=LCP2 PE=1 SV=1 | LCP2 | 0.1569 | 0.0494 |
| Q9NZC3 | Glycerophosphodiester phosphodiesterase 1 OS=Homo sapiens OX=9606 GN=GDE1 PE=1 SV=1 | GDE1 | 0.1573 | 0.0011 |
| Q9BPV8 | P2Y purinoceptor 13 OS=Homo sapiens OX=9606 GN=P2RY13 PE=1 SV=3 | P2RY13 | 0.1635 | 0.0039 |
| P05186 | Alkaline phosphatase, tissue-nonspecific isozyme OS=Homo sapiens OX=9606 GN=ALPL PE=1 SV=4 | ALPL | 0.1641 | 0.0327 |
| P52790 | Hexokinase-3 OS=Homo sapiens OX=9606 GN=HK3 PE=1 SV=2 | HK3 | 0.1645 | 0.0500 |
| P98066 | Tumor necrosis factor-inducible gene 6 protein OS=Homo sapiens OX=9606 GN=TNFAIP6 PE=1 SV=2 | TNFAIP6 | 0.1741 | 0.0424 |
| P21730 | C5a anaphylatoxin chemotactic receptor 1 OS=Homo sapiens OX=9606 GN=C5AR1 PE=1 SV=2 | C5AR1 | 0.1886 | 0.0040 |
| P22894 | Neutrophil collagenase OS=Homo sapiens OX=9606 GN=MMP8 PE=1 SV=1 | MMP8 | 0.1888 | 0.0397 |
| Q8IWV8 | E3 ubiquitin-protein ligase UBR2 OS=Homo sapiens OX=9606 GN=UBR2 PE=1 SV=1 | UBR2 | 0.1891 | 0.0099 |
| P78325 | Disintegrin and metalloproteinase domain-containing protein 8 OS=Homo sapiens OX=9606 GN=ADAM8 PE=1 SV=2 | ADAM8 | 0.1903 | 0.0201 |
| Q6ZMI0 | Protein phosphatase 1 regulatory subunit 21 OS=Homo sapiens OX=9606 GN=PPP1R21 PE=1 SV=1 | PPP1R21 | 0.1926 | 0.0084 |
| Q9BXF6 | Rab11 family-interacting protein 5 OS=Homo sapiens OX=9606 GN=RAB11FIP5 PE=1 SV=1 | RAB11FIP5 | 0.1944 | 0.0330 |
| P11215 | Integrin alpha-M OS=Homo sapiens OX=9606 GN=ITGAM PE=1 SV=2 | ITGAM | 0.2004 | 0.0228 |
| O75923 | Dysferlin OS=Homo sapiens OX=9606 GN=DYSF PE=1 SV=1 | DYSF | 0.2102 | 0.0154 |
| P20292 | Arachidonate 5-lipoxygenase-activating protein OS=Homo sapiens OX=9606 GN=ALOX5AP PE=1 SV=2 | ALOX5AP | 0.2107 | 0.0199 |
| P48740 | Mannan-binding lectin serine protease 1 OS=Homo sapiens OX=9606 GN=MASP1 PE=1 SV=3 | MASP1 | 0.2143 | 0.0378 |
| Q9BZI7 | Regulator of nonsense transcripts 3B OS=Homo sapiens OX=9606 GN=UPF3B PE=1 SV=1 | UPF3B | 0.2147 | 0.0281 |
| P20132 | L-serine dehydratase/L-threonine deaminase OS=Homo sapiens OX=9606 GN=SDS PE=1 SV=2 | SDS | 0.2155 | 0.0301 |
| Q71RC2 | La-related protein 4 OS=Homo sapiens OX=9606 GN=LARP4 PE=1 SV=3 | LARP4 | 0.2205 | 0.0095 |
| P05107 | Integrin beta-2 OS=Homo sapiens OX=9606 GN=ITGB2 PE=1 SV=2 | ITGB2 | 0.2247 | 0.0132 |
| Q9UBS3 | DnaJ homolog subfamily B member 9 OS=Homo sapiens OX=9606 GN=DNAJB9 PE=1 SV=1 | DNAJB9 | 0.2287 | 0.0267 |
| P19878 | Neutrophil cytosol factor 2 OS=Homo sapiens OX=9606 GN=NCF2 PE=1 SV=2 | NCF2 | 0.2406 | 0.0013 |
| Q9Y6A5 | Transforming acidic coiled-coil-containing protein 3 OS=Homo sapiens OX=9606 GN=TACC3 PE=1 SV=1 | TACC3 | 0.2408 | 0.0206 |
| P27105 | Stomatin OS=Homo sapiens OX=9606 GN=STOM PE=1 SV=3 | STOM | 0.2431 | 0.0155 |
| O95757 | Heat shock 70 kDa protein 4L OS=Homo sapiens OX=9606 GN=HSPA4L PE=1 SV=3 | HSPA4L | 0.2442 | 0.0052 |
| Q15386 | Ubiquitin-protein ligase E3C OS=Homo sapiens OX=9606 GN=UBE3C PE=1 SV=3 | UBE3C | 0.2448 | 0.0318 |
| Q96KN4 | Protein LRATD1 OS=Homo sapiens OX=9606 GN=LRATD1 PE=1 SV=2 | LRATD1 | 0.253 | 0.0450 |
| Q7L591 | Docking protein 3 OS=Homo sapiens OX=9606 GN=DOK3 PE=1 SV=2 | DOK3 | 0.2538 | 0.0360 |
| O43166 | Signal-induced proliferation-associated 1-like protein 1 OS=Homo sapiens OX=9606 GN=SIPA1L1 PE=1 SV=4 | SIPA1L1 | 0.2652 | 0.0233 |
| O75781 | Paralemmin-1 OS=Homo sapiens OX=9606 GN=PALM PE=1 SV=2 | PALM | 0.2663 | 0.0345 |
| Q687X5 | Metalloreductase STEAP4 OS=Homo sapiens OX=9606 GN=STEAP4 PE=1 SV=1 | STEAP4 | 0.2667 | 0.0319 |
| Q9NX00 | Transmembrane protein 160 OS=Homo sapiens OX=9606 GN=TMEM160 PE=1 SV=1 | TMEM160 | 0.2693 | 0.0106 |
| Q8IV38 | Ankyrin repeat and MYND domain-containing protein 2 OS=Homo sapiens OX=9606 GN=ANKMY2 PE=1 SV=1 | ANKMY2 | 0.2717 | 0.0420 |
| Q8IX19 | Mast cell-expressed membrane protein 1 OS=Homo sapiens OX=9606 GN=MCEMP1 PE=1 SV=1 | MCEMP1 | 0.2742 | 0.0141 |
| Q9UPR0 | Inactive phospholipase C-like protein 2 OS=Homo sapiens OX=9606 GN=PLCL2 PE=1 SV=2 | PLCL2 | 0.2751 | 0.0473 |
| Q7Z403 | Transmembrane channel-like protein 6 OS=Homo sapiens OX=9606 GN=TMC6 PE=1 SV=2 | TMC6 | 0.2827 | 0.0038 |
| P08631 | Tyrosine-protein kinase HCK OS=Homo sapiens OX=9606 GN=HCK PE=1 SV=5 | HCK | 0.2899 | 0.0473 |
| Q9ULH1 | Arf-GAP with SH3 domain, ANK repeat and PH domain-containing protein 1 OS=Homo sapiens OX=9606 GN=ASAP1 PE=1 SV=4 | ASAP1 | 0.2926 | 0.0207 |
| Q10588 | ADP-ribosyl cyclase/cyclic ADP-ribose hydrolase 2 OS=Homo sapiens OX=9606 GN=BST1 PE=1 SV=2 | BST1 | 0.2944 | 0.0358 |
| O43818 | U3 small nucleolar RNA-interacting protein 2 OS=Homo sapiens OX=9606 GN=RRP9 PE=1 SV=1 | RRP9 | 0.3001 | 0.0175 |
| O60462 | Neuropilin-2 OS=Homo sapiens OX=9606 GN=NRP2 PE=1 SV=3 | NRP2 | 0.3048 | 0.0387 |
| Q9NS00 | Glycoprotein-N-acetylgalactosamine 3-beta-galactosyltransferase 1 OS=Homo sapiens OX=9606 GN=C1GALT1 PE=1 SV=1 | C1GALT1 | 0.3059 | 0.0088 |
| Q6NUQ1 | RAD50-interacting protein 1 OS=Homo sapiens OX=9606 GN=RINT1 PE=1 SV=1 | RINT1 | 0.306 | 0.0291 |
| P26927 | Hepatocyte growth factor-like protein OS=Homo sapiens OX=9606 GN=MST1 PE=1 SV=2 | MST1 | 0.3068 | 0.0081 |
| Q7L5N7 | Lysophosphatidylcholine acyltransferase 2 OS=Homo sapiens OX=9606 GN=LPCAT2 PE=1 SV=1 | LPCAT2 | 0.312 | 0.0270 |
| P12314 | High affinity immunoglobulin gamma Fc receptor I OS=Homo sapiens OX=9606 GN=FCGR1A PE=1 SV=2 | FCGR1A | 0.3161 | 0.0092 |
| P20718 | Granzyme H OS=Homo sapiens OX=9606 GN=GZMH PE=1 SV=1 | GZMH | 0.3165 | 0.0082 |
| P00747 | Plasminogen OS=Homo sapiens OX=9606 GN=PLG PE=1 SV=2 | PLG | 0.3229 | 0.0339 |
| P42229 | Signal transducer and activator of transcription 5A OS=Homo sapiens OX=9606 GN=STAT5A PE=1 SV=1 | STAT5A | 0.3239 | 0.0153 |
| Q9H7M9 | V-type immunoglobulin domain-containing suppressor of T-cell activation OS=Homo sapiens OX=9606 GN=VSIR PE=1 SV=3 | VSIR | 0.3284 | 0.0442 |
| P49795 | Regulator of G-protein signaling 19 OS=Homo sapiens OX=9606 GN=RGS19 PE=1 SV=1 | RGS19 | 0.3286 | 0.0232 |
| Q92985 | Interferon regulatory factor 7 OS=Homo sapiens OX=9606 GN=IRF7 PE=1 SV=2 | IRF7 | 0.3303 | 0.0313 |
| P42345 | Serine/threonine-protein kinase mTOR OS=Homo sapiens OX=9606 GN=MTOR PE=1 SV=1 | MTOR | 0.3327 | 0.0260 |
| P04839 | Cytochrome b-245 heavy chain OS=Homo sapiens OX=9606 GN=CYBB PE=1 SV=2 | CYBB | 0.3352 | 0.0240 |
| P04114 | Apolipoprotein B-100 OS=Homo sapiens OX=9606 GN=APOB PE=1 SV=2 | APOB | 0.3359 | 0.0442 |
| P04003 | C4b-binding protein alpha chain OS=Homo sapiens OX=9606 GN=C4BPA PE=1 SV=2 | C4BPA | 0.3426 | 0.0171 |
| Q8TEV9 | Guanine nucleotide exchange protein SMCR8 OS=Homo sapiens OX=9606 GN=SMCR8 PE=1 SV=2 | SMCR8 | 0.3427 | 0.0082 |
| P12318 | Low affinity immunoglobulin gamma Fc region receptor II-a OS=Homo sapiens OX=9606 GN=FCGR2A PE=1 SV=4 | FCGR2A | 0.3438 | 0.0450 |
| P09619 | Platelet-derived growth factor receptor beta OS=Homo sapiens OX=9606 GN=PDGFRB PE=1 SV=1 | PDGFRB | 0.3511 | 0.0098 |
| Q15654 | Thyroid receptor-interacting protein 6 OS=Homo sapiens OX=9606 GN=TRIP6 PE=1 SV=3 | TRIP6 | 0.3582 | 0.0485 |
| O95544 | NAD kinase OS=Homo sapiens OX=9606 GN=NADK PE=1 SV=1 | NADK | 0.3584 | 0.0128 |
| O43566 | Regulator of G-protein signaling 14 OS=Homo sapiens OX=9606 GN=RGS14 PE=1 SV=4 | RGS14 | 0.3594 | 0.0110 |
| Q6ZVF9 | G protein-regulated inducer of neurite outgrowth 3 OS=Homo sapiens OX=9606 GN=GPRIN3 PE=2 SV=2 | GPRIN3 | 0.3624 | 0.0232 |
| P42226 | Signal transducer and activator of transcription 6 OS=Homo sapiens OX=9606 GN=STAT6 PE=1 SV=1 | STAT6 | 0.3671 | 0.0137 |
| Q8N0X7 | Spartin OS=Homo sapiens OX=9606 GN=SPART PE=1 SV=1 | SPART | 0.3702 | 0.0153 |
| Q8NG11 | Tetraspanin-14 OS=Homo sapiens OX=9606 GN=TSPAN14 PE=1 SV=1 | TSPAN14 | 0.3721 | 0.0384 |
| Q13488 | V-type proton ATPase 116 kDa subunit a3 OS=Homo sapiens OX=9606 GN=TCIRG1 PE=1 SV=3 | TCIRG1 | 0.3756 | 0.0317 |
| Q9UHR5 | SAP30-binding protein OS=Homo sapiens OX=9606 GN=SAP30BP PE=1 SV=1 | SAP30BP | 0.3795 | 0.0462 |
| Q8WVN6 | Secreted and transmembrane protein 1 OS=Homo sapiens OX=9606 GN=SECTM1 PE=1 SV=2 | SECTM1 | 0.3801 | 0.0187 |
| P28039 | Acyloxyacyl hydrolase OS=Homo sapiens OX=9606 GN=AOAH PE=1 SV=1 | AOAH | 0.3898 | 0.0009 |
| P48436 | Transcription factor SOX-9 OS=Homo sapiens OX=9606 GN=SOX9 PE=1 SV=1 | SOX9 | 0.39 | 0.0149 |
| Q9ULX9 | Transcription factor MafF OS=Homo sapiens OX=9606 GN=MAFF PE=1 SV=2 | MAFF | 0.3982 | 0.0188 |
| Q9H7E9 | UPF0488 protein C8orf33 OS=Homo sapiens OX=9606 GN=C8orf33 PE=1 SV=1 | C8orf33 | 0.4009 | 0.0414 |
| Q13751 | Laminin subunit beta-3 OS=Homo sapiens OX=9606 GN=LAMB3 PE=1 SV=1 | LAMB3 | 0.4035 | 0.0130 |
| P16150 | Leukosialin OS=Homo sapiens OX=9606 GN=SPN PE=1 SV=1 | SPN | 0.4058 | 0.0470 |
| P46013 | Proliferation marker protein Ki-67 OS=Homo sapiens OX=9606 GN=MKI67 PE=1 SV=2 | MKI67 | 0.4095 | 0.0446 |
| P35354 | Prostaglandin G/H synthase 2 OS=Homo sapiens OX=9606 GN=PTGS2 PE=1 SV=2 | PTGS2 | 0.4115 | 0.0278 |
| P62136 | Serine/threonine-protein phosphatase PP1-alpha catalytic subunit OS=Homo sapiens OX=9606 GN=PPP1CA PE=1 SV=1 | PPP1CA | 0.4134 | 0.0202 |
| Q8NC42 | E3 ubiquitin-protein ligase RNF149 OS=Homo sapiens OX=9606 GN=RNF149 PE=2 SV=2 | RNF149 | 0.4158 | 0.0481 |
| Q9Y2X3 | Nucleolar protein 58 OS=Homo sapiens OX=9606 GN=NOP58 PE=1 SV=1 | NOP58 | 0.419 | 0.0496 |
| Q14244 | Ensconsin OS=Homo sapiens OX=9606 GN=MAP7 PE=1 SV=1 | MAP7 | 0.4281 | 0.0213 |
| Q6Y288 | Beta-1,3-glucosyltransferase OS=Homo sapiens OX=9606 GN=B3GLCT PE=1 SV=2 | B3GLCT | 0.4349 | 0.0146 |
| A6NC98 | Coiled-coil domain-containing protein 88B OS=Homo sapiens OX=9606 GN=CCDC88B PE=1 SV=1 | CCDC88B | 0.4357 | 0.0182 |
| Q9UKA4 | A-kinase anchor protein 11 OS=Homo sapiens OX=9606 GN=AKAP11 PE=1 SV=1 | AKAP11 | 0.4402 | 0.0306 |
| Q99638 | Cell cycle checkpoint control protein RAD9A OS=Homo sapiens OX=9606 GN=RAD9A PE=1 SV=1 | RAD9A | 0.4402 | 0.0200 |
| Q15075 | Early endosome antigen 1 OS=Homo sapiens OX=9606 GN=EEA1 PE=1 SV=2 | EEA1 | 0.4436 | 0.0115 |
| A6NI72 | Putative neutrophil cytosol factor 1B OS=Homo sapiens OX=9606 GN=NCF1B PE=5 SV=2 | NCF1B | 0.4585 | 0.0156 |
| O15162 | Phospholipid scramblase 1 OS=Homo sapiens OX=9606 GN=PLSCR1 PE=1 SV=1 | PLSCR1 | 0.4586 | 0.0038 |
| Q05209 | Tyrosine-protein phosphatase non-receptor type 12 OS=Homo sapiens OX=9606 GN=PTPN12 PE=1 SV=3 | PTPN12 | 0.4589 | 0.0414 |
| Q9UM07 | Protein-arginine deiminase type-4 OS=Homo sapiens OX=9606 GN=PADI4 PE=1 SV=2 | PADI4 | 0.4607 | 0.0258 |
| Q96EQ0 | Small glutamine-rich tetratricopeptide repeat-containing protein beta OS=Homo sapiens OX=9606 GN=SGTB PE=1 SV=1 | SGTB | 0.4647 | 0.0111 |
| P04083 | Annexin A1 OS=Homo sapiens OX=9606 GN=ANXA1 PE=1 SV=2 | ANXA1 | 0.4745 | 0.0378 |
| P32942 | Intercellular adhesion molecule 3 OS=Homo sapiens OX=9606 GN=ICAM3 PE=1 SV=2 | ICAM3 | 0.482 | 0.0476 |
| Q8NFZ5 | TNFAIP3-interacting protein 2 OS=Homo sapiens OX=9606 GN=TNIP2 PE=1 SV=1 | TNIP2 | 0.4823 | 0.0457 |
| O75410 | Transforming acidic coiled-coil-containing protein 1 OS=Homo sapiens OX=9606 GN=TACC1 PE=1 SV=2 | TACC1 | 0.4839 | 0.0397 |
| Q8TDB6 | E3 ubiquitin-protein ligase DTX3L OS=Homo sapiens OX=9606 GN=DTX3L PE=1 SV=1 | DTX3L | 0.4843 | 0.0271 |
| Q13043 | Serine/threonine-protein kinase 4 OS=Homo sapiens OX=9606 GN=STK4 PE=1 SV=2 | STK4 | 0.4866 | 0.0330 |
| Q68DK2 | Zinc finger FYVE domain-containing protein 26 OS=Homo sapiens OX=9606 GN=ZFYVE26 PE=1 SV=3 | ZFYVE26 | 0.4937 | 0.0273 |
| Q9UQE7 | Structural maintenance of chromosomes protein 3 OS=Homo sapiens OX=9606 GN=SMC3 PE=1 SV=2 | SMC3 | 0.4993 | 0.0173 |
| P06681 | Complement C2 OS=Homo sapiens OX=9606 GN=C2 PE=1 SV=2 | C2 | 0.5011 | 0.0124 |
| Q9NV96 | Cell cycle control protein 50A OS=Homo sapiens OX=9606 GN=TMEM30A PE=1 SV=1 | TMEM30A | 0.5186 | 0.0295 |
| P39059 | Collagen alpha-1(XV) chain OS=Homo sapiens OX=9606 GN=COL15A1 PE=1 SV=2 | COL15A1 | 0.5305 | 0.0401 |
| P00734 | Prothrombin OS=Homo sapiens OX=9606 GN=F2 PE=1 SV=2 | F2 | 0.5327 | 0.0371 |
| P53671 | LIM domain kinase 2 OS=Homo sapiens OX=9606 GN=LIMK2 PE=1 SV=1 | LIMK2 | 0.5411 | 0.0238 |
| Q13948 | Protein CASP OS=Homo sapiens OX=9606 GN=CUX1 PE=1 SV=2 | CUX1 | 0.5493 | 0.0233 |
| P39748 | Flap endonuclease 1 OS=Homo sapiens OX=9606 GN=FEN1 PE=1 SV=1 | FEN1 | 0.5536 | 0.0259 |
| P54259 | Atrophin-1 OS=Homo sapiens OX=9606 GN=ATN1 PE=1 SV=3 | ATN1 | 0.5537 | 0.0484 |
| P15153 | Ras-related C3 botulinum toxin substrate 2 OS=Homo sapiens OX=9606 GN=RAC2 PE=1 SV=1 | RAC2 | 0.5693 | 0.0408 |
| P54252 | Ataxin-3 OS=Homo sapiens OX=9606 GN=ATXN3 PE=1 SV=5 | ATXN3 | 0.5814 | 0.0430 |
| A6NKD9 | Coiled-coil domain-containing protein 85C OS=Homo sapiens OX=9606 GN=CCDC85C PE=1 SV=1 | CCDC85C | 0.5836 | 0.0495 |
| P13995 | Bifunctional methylenetetrahydrofolate dehydrogenase/cyclohydrolase, mitochondrial OS=Homo sapiens OX=9606 GN=MTHFD2 PE=1 SV=2 | MTHFD2 | 0.5869 | 0.0167 |
| Q96RT7 | Gamma-tubulin complex component 6 OS=Homo sapiens OX=9606 GN=TUBGCP6 PE=1 SV=3 | TUBGCP6 | 0.5878 | 0.0332 |
| P19525 | Interferon-induced, double-stranded RNA-activated protein kinase OS=Homo sapiens OX=9606 GN=EIF2AK2 PE=1 SV=2 | EIF2AK2 | 0.5889 | 0.0370 |
| P30622 | CAP-Gly domain-containing linker protein 1 OS=Homo sapiens OX=9606 GN=CLIP1 PE=1 SV=2 | CLIP1 | 0.5978 | 0.0301 |
| Q9Y2H6 | Fibronectin type-III domain-containing protein 3A OS=Homo sapiens OX=9606 GN=FNDC3A PE=1 SV=4 | FNDC3A | 0.6046 | 0.0465 |
| Q7L523 | Ras-related GTP-binding protein A OS=Homo sapiens OX=9606 GN=RRAGA PE=1 SV=1 | RRAGA | 0.6064 | 0.0270 |
| P29401 | Transketolase OS=Homo sapiens OX=9606 GN=TKT PE=1 SV=3 | TKT | 0.6147 | 0.0034 |
| Q01804 | OTU domain-containing protein 4 OS=Homo sapiens OX=9606 GN=OTUD4 PE=1 SV=4 | OTUD4 | 0.6226 | 0.0173 |
| Q15052 | Rho guanine nucleotide exchange factor 6 OS=Homo sapiens OX=9606 GN=ARHGEF6 PE=1 SV=2 | ARHGEF6 | 0.6264 | 0.0363 |
| P30511 | HLA class I histocompatibility antigen, alpha chain F OS=Homo sapiens OX=9606 GN=HLA-F PE=1 SV=3 | HLA-F | 0.6288 | 0.0253 |
| Q9HDC9 | Adipocyte plasma membrane-associated protein OS=Homo sapiens OX=9606 GN=APMAP PE=1 SV=2 | APMAP | 0.6313 | 0.0360 |
| Q9HD26 | Golgi-associated PDZ and coiled-coil motif-containing protein OS=Homo sapiens OX=9606 GN=GOPC PE=1 SV=1 | GOPC | 0.6339 | 0.0390 |
| Q92925 | SWI/SNF-related matrix-associated actin-dependent regulator of chromatin subfamily D member 2 OS=Homo sapiens OX=9606 GN=SMARCD2 PE=1 SV=3 | SMARCD2 | 0.6351 | 0.0490 |
| Q6ZNJ1 | Neurobeachin-like protein 2 OS=Homo sapiens OX=9606 GN=NBEAL2 PE=1 SV=2 | NBEAL2 | 0.638 | 0.0326 |
| Q5JRA6 | Transport and Golgi organization protein 1 homolog OS=Homo sapiens OX=9606 GN=MIA3 PE=1 SV=1 | MIA3 | 0.6414 | 0.0474 |
| Q5T200 | Zinc finger CCCH domain-containing protein 13 OS=Homo sapiens OX=9606 GN=ZC3H13 PE=1 SV=1 | ZC3H13 | 0.6537 | 0.0279 |
| Up-regulated proteins | | | | |
| Q8WWA0 | Intelectin-1 OS=Homo sapiens OX=9606 GN=ITLN1 PE=1 SV=1 | ITLN1 | 51.9635 | 0.0066 |
| Q14002 | Carcinoembryonic antigen-related cell adhesion molecule 7 OS=Homo sapiens OX=9606 GN=CEACAM7 PE=1 SV=1 | CEACAM7 | 21.8368 | 0.0118 |
| Q9HBY8 | Serine/threonine-protein kinase Sgk2 OS=Homo sapiens OX=9606 GN=SGK2 PE=1 SV=1 | SGK2 | 21.4752 | 0.0282 |
| Q6ZSS7 | Major facilitator superfamily domain-containing protein 6 OS=Homo sapiens OX=9606 GN=MFSD6 PE=1 SV=2 | MFSD6 | 16.5788 | 0.0025 |
| O00585 | C-C motif chemokine 21 OS=Homo sapiens OX=9606 GN=CCL21 PE=1 SV=1 | CCL21 | 12.1642 | 0.0455 |
| P34096 | Ribonuclease 4 OS=Homo sapiens OX=9606 GN=RNASE4 PE=1 SV=3 | RNASE4 | 8.2467 | 0.0327 |
| P04066 | Tissue alpha-L-fucosidase OS=Homo sapiens OX=9606 GN=FUCA1 PE=1 SV=4 | FUCA1 | 7.9789 | 0.0086 |
| O60218 | Aldo-keto reductase family 1 member B10 OS=Homo sapiens OX=9606 GN=AKR1B10 PE=1 SV=2 | AKR1B10 | 7.7816 | 0.0037 |
| Q9BXN1 | Asporin OS=Homo sapiens OX=9606 GN=ASPN PE=1 SV=2 | ASPN | 6.945 | 0.0089 |
| Q6JQN1 | Acyl-CoA dehydrogenase family member 10 OS=Homo sapiens OX=9606 GN=ACAD10 PE=1 SV=1 | ACAD10 | 6.1302 | 0.0038 |
| Q9C002 | Normal mucosa of esophagus-specific gene 1 protein OS=Homo sapiens OX=9606 GN=NMES1 PE=2 SV=1 | NMES1 | 6.1046 | 0.0327 |
| Q495W5 | Alpha-(1,3)-fucosyltransferase 11 OS=Homo sapiens OX=9606 GN=FUT11 PE=1 SV=1 | FUT11 | 6.0461 | 0.0293 |
| P50225 | Sulfotransferase 1A1 OS=Homo sapiens OX=9606 GN=SULT1A1 PE=1 SV=3 | SULT1A1 | 5.9883 | 0.0365 |
| Q9BW66 | Cyclin-dependent kinase 2-interacting protein OS=Homo sapiens OX=9606 GN=CINP PE=1 SV=1 | CINP | 5.3925 | 0.0168 |
| P0DMM9 | Sulfotransferase 1A3 OS=Homo sapiens OX=9606 GN=SULT1A3 PE=1 SV=1 | SULT1A3 | 5.2444 | 0.0285 |
| O43674 | NADH dehydrogenase [ubiquinone] 1 beta subcomplex subunit 5, mitochondrial OS=Homo sapiens OX=9606 GN=NDUFB5 PE=1 SV=1 | NDUFB5 | 5.2425 | 0.0397 |
| O75489 | NADH dehydrogenase [ubiquinone] iron-sulfur protein 3, mitochondrial OS=Homo sapiens OX=9606 GN=NDUFS3 PE=1 SV=1 | NDUFS3 | 4.9635 | 0.0231 |
| Q9BSU1 | UPF0183 protein C16orf70 OS=Homo sapiens OX=9606 GN=C16orf70 PE=1 SV=1 | C16orf70 | 4.9635 | 0.0109 |
| Q6NUM9 | All-trans-retinol 13,14-reductase OS=Homo sapiens OX=9606 GN=RETSAT PE=1 SV=2 | RETSAT | 4.8066 | 0.0208 |
| Q53GD3 | Choline transporter-like protein 4 OS=Homo sapiens OX=9606 GN=SLC44A4 PE=1 SV=2 | SLC44A4 | 4.6964 | 0.0236 |
| P21741 | Midkine OS=Homo sapiens OX=9606 GN=MDK PE=1 SV=1 | MDK | 4.3812 | 0.0344 |
| Q8NI37 | Protein phosphatase PTC7 homolog OS=Homo sapiens OX=9606 GN=PPTC7 PE=1 SV=1 | PPTC7 | 4.1688 | 0.0297 |
| Q9Y244 | Proteasome maturation protein OS=Homo sapiens OX=9606 GN=POMP PE=1 SV=1 | POMP | 4.045 | 0.0462 |
| P13716 | Delta-aminolevulinic acid dehydratase OS=Homo sapiens OX=9606 GN=ALAD PE=1 SV=1 | ALAD | 3.9186 | 0.0473 |
| P14207 | Folate receptor beta OS=Homo sapiens OX=9606 GN=FOLR2 PE=1 SV=4 | FOLR2 | 3.65 | 0.0285 |
| O75438 | NADH dehydrogenase [ubiquinone] 1 beta subcomplex subunit 1 OS=Homo sapiens OX=9606 GN=NDUFB1 PE=1 SV=1 | NDUFB1 | 3.6123 | 0.0441 |
| Q14012 | Calcium/calmodulin-dependent protein kinase type 1 OS=Homo sapiens OX=9606 GN=CAMK1 PE=1 SV=1 | CAMK1 | 3.6067 | 0.0237 |
| O14521 | Succinate dehydrogenase [ubiquinone] cytochrome b small subunit, mitochondrial OS=Homo sapiens OX=9606 GN=SDHD PE=1 SV=1 | SDHD | 3.5035 | 0.0036 |
| P20290 | Transcription factor BTF3 OS=Homo sapiens OX=9606 GN=BTF3 PE=1 SV=1 | BTF3 | 3.5025 | 0.0036 |
| Q9UI30 | Multifunctional methyltransferase subunit TRM112-like protein OS=Homo sapiens OX=9606 GN=TRMT112 PE=1 SV=1 | TRMT112 | 3.4326 | 0.0050 |
| O43150 | Arf-GAP with SH3 domain, ANK repeat and PH domain-containing protein 2 OS=Homo sapiens OX=9606 GN=ASAP2 PE=1 SV=3 | ASAP2 | 3.3258 | 0.0464 |
| P00750 | Tissue-type plasminogen activator OS=Homo sapiens OX=9606 GN=PLAT PE=1 SV=1 | PLAT | 3.1687 | 0.0192 |
| Q9NZD2 | Glycolipid transfer protein OS=Homo sapiens OX=9606 GN=GLTP PE=1 SV=3 | GLTP | 3.1465 | 0.0302 |
| Q9UBC5 | Unconventional myosin-Ia OS=Homo sapiens OX=9606 GN=MYO1A PE=1 SV=1 | MYO1A | 3.1448 | 0.0441 |
| Q9HCY8 | Protein S100-A14 OS=Homo sapiens OX=9606 GN=S100A14 PE=1 SV=1 | S100A14 | 3.1098 | 0.0300 |
| O15195 | Villin-like protein OS=Homo sapiens OX=9606 GN=VILL PE=2 SV=3 | VILL | 3.0603 | 0.0444 |
| Q9BTD8 | RNA-binding protein 42 OS=Homo sapiens OX=9606 GN=RBM42 PE=1 SV=1 | RBM42 | 2.9986 | 0.0269 |
| O43665 | Regulator of G-protein signaling 10 OS=Homo sapiens OX=9606 GN=RGS10 PE=1 SV=3 | RGS10 | 2.9755 | 0.0455 |
| O94967 | WD repeat-containing protein 47 OS=Homo sapiens OX=9606 GN=WDR47 PE=1 SV=1 | WDR47 | 2.9704 | 0.0019 |
| P18440 | Arylamine N-acetyltransferase 1 OS=Homo sapiens OX=9606 GN=NAT1 PE=1 SV=2 | NAT1 | 2.796 | 0.0172 |
| O75376 | Nuclear receptor corepressor 1 OS=Homo sapiens OX=9606 GN=NCOR1 PE=1 SV=2 | NCOR1 | 2.7834 | 0.0122 |
| Q9BPW9 | Dehydrogenase/reductase SDR family member 9 OS=Homo sapiens OX=9606 GN=DHRS9 PE=1 SV=1 | DHRS9 | 2.7817 | 0.0158 |
| Q8WV74 | Nucleoside diphosphate-linked moiety X motif 8 OS=Homo sapiens OX=9606 GN=NUDT8 PE=1 SV=2 | NUDT8 | 2.7706 | 0.0406 |
| Q86U28 | Iron-sulfur cluster assembly 2 homolog, mitochondrial OS=Homo sapiens OX=9606 GN=ISCA2 PE=1 SV=2 | ISCA2 | 2.7662 | 0.0022 |
| Q53EL6 | Programmed cell death protein 4 OS=Homo sapiens OX=9606 GN=PDCD4 PE=1 SV=2 | PDCD4 | 2.7471 | 0.0476 |
| Q8N5M9 | Protein jagunal homolog 1 OS=Homo sapiens OX=9606 GN=JAGN1 PE=1 SV=1 | JAGN1 | 2.7436 | 0.0205 |
| Q9UBX5 | Fibulin-5 OS=Homo sapiens OX=9606 GN=FBLN5 PE=1 SV=1 | FBLN5 | 2.7384 | 0.0481 |
| Q8TE02 | Elongator complex protein 5 OS=Homo sapiens OX=9606 GN=ELP5 PE=1 SV=2 | ELP5 | 2.7373 | 0.0340 |
| Q9UFG5 | UPF0449 protein C19orf25 OS=Homo sapiens OX=9606 GN=C19orf25 PE=1 SV=2 | C19orf25 | 2.6827 | 0.0322 |
| Q86U42 | Polyadenylate-binding protein 2 OS=Homo sapiens OX=9606 GN=PABPN1 PE=1 SV=3 | PABPN1 | 2.6817 | 0.0219 |
| Q96F85 | CB1 cannabinoid receptor-interacting protein 1 OS=Homo sapiens OX=9606 GN=CNRIP1 PE=1 SV=1 | CNRIP1 | 2.6743 | 0.0366 |
| Q9UQ03 | Coronin-2B OS=Homo sapiens OX=9606 GN=CORO2B PE=1 SV=4 | CORO2B | 2.6514 | 0.0277 |
| P19404 | NADH dehydrogenase [ubiquinone] flavoprotein 2, mitochondrial OS=Homo sapiens OX=9606 GN=NDUFV2 PE=1 SV=2 | NDUFV2 | 2.6164 | 0.0350 |
| Q9NRD5 | PRKCA-binding protein OS=Homo sapiens OX=9606 GN=PICK1 PE=1 SV=2 | PICK1 | 2.5955 | 0.0436 |
| Q9UNT1 | Rab-like protein 2B OS=Homo sapiens OX=9606 GN=RABL2B PE=1 SV=1 | RABL2B | 2.5745 | 0.0029 |
| Q9UL12 | Sarcosine dehydrogenase, mitochondrial OS=Homo sapiens OX=9606 GN=SARDH PE=1 SV=1 | SARDH | 2.5145 | 0.0453 |
| P22694 | cAMP-dependent protein kinase catalytic subunit beta OS=Homo sapiens OX=9606 GN=PRKACB PE=1 SV=2 | PRKACB | 2.5081 | 0.0418 |
| Q9BSF0 | Small membrane A-kinase anchor protein OS=Homo sapiens OX=9606 GN=C2orf88 PE=1 SV=2 | C2orf88 | 2.434 | 0.0383 |
| Q92796 | Disks large homolog 3 OS=Homo sapiens OX=9606 GN=DLG3 PE=1 SV=2 | DLG3 | 2.4307 | 0.0457 |
| Q7L7V1 | Putative pre-mRNA-splicing factor ATP-dependent RNA helicase DHX32 OS=Homo sapiens OX=9606 GN=DHX32 PE=1 SV=1 | DHX32 | 2.4008 | 0.0366 |
| O43520 | Phospholipid-transporting ATPase IC OS=Homo sapiens OX=9606 GN=ATP8B1 PE=1 SV=3 | ATP8B1 | 2.3471 | 0.0362 |
| Q8TBX8 | Phosphatidylinositol 5-phosphate 4-kinase type-2 gamma OS=Homo sapiens OX=9606 GN=PIP4K2C PE=1 SV=3 | PIP4K2C | 2.3428 | 0.0091 |
| Q14651 | Plastin-1 OS=Homo sapiens OX=9606 GN=PLS1 PE=1 SV=2 | PLS1 | 2.3138 | 0.0028 |
| O95340 | Bifunctional 3'-phosphoadenosine 5'-phosphosulfate synthase 2 OS=Homo sapiens OX=9606 GN=PAPSS2 PE=1 SV=2 | PAPSS2 | 2.2986 | 0.0019 |
| O95671 | Probable bifunctional dTTP/UTP pyrophosphatase/methyltransferase protein OS=Homo sapiens OX=9606 GN=ASMTL PE=1 SV=3 | ASMTL | 2.2652 | 0.0448 |
| Q9H469 | F-box/LRR-repeat protein 15 OS=Homo sapiens OX=9606 GN=FBXL15 PE=1 SV=2 | FBXL15 | 2.1426 | 0.0024 |
| Q9Y3D3 | 28S ribosomal protein S16, mitochondrial OS=Homo sapiens OX=9606 GN=MRPS16 PE=1 SV=1 | MRPS16 | 2.1139 | 0.0208 |
| Q15121 | Astrocytic phosphoprotein PEA-15 OS=Homo sapiens OX=9606 GN=PEA15 PE=1 SV=2 | PEA15 | 2.0889 | 0.0423 |
| Q5T5U3 | Rho GTPase-activating protein 21 OS=Homo sapiens OX=9606 GN=ARHGAP21 PE=1 SV=2 | ARHGAP21 | 2.0264 | 0.0331 |
| P43251 | Biotinidase OS=Homo sapiens OX=9606 GN=BTD PE=1 SV=2 | BTD | 2.0206 | 0.0341 |
| Q9NR19 | Acetyl-coenzyme A synthetase, cytoplasmic OS=Homo sapiens OX=9606 GN=ACSS2 PE=1 SV=1 | ACSS2 | 2.0186 | 0.0258 |
| P23610 | 40-kDa huntingtin-associated protein OS=Homo sapiens OX=9606 GN=F8A1 PE=1 SV=2 | F8A1 | 1.9897 | 0.0426 |
| P37108 | Signal recognition particle 14 kDa protein OS=Homo sapiens OX=9606 GN=SRP14 PE=1 SV=2 | SRP14 | 1.9801 | 0.0038 |
| Q92508 | Piezo-type mechanosensitive ion channel component 1 OS=Homo sapiens OX=9606 GN=PIEZO1 PE=1 SV=4 | PIEZO1 | 1.9789 | 0.0170 |
| Q9P013 | Spliceosome-associated protein CWC15 homolog OS=Homo sapiens OX=9606 GN=CWC15 PE=1 SV=2 | CWC15 | 1.9703 | 0.0247 |
| Q9BPX5 | Actin-related protein 2/3 complex subunit 5-like protein OS=Homo sapiens OX=9606 GN=ARPC5L PE=1 SV=1 | ARPC5L | 1.97 | 0.0247 |
| P61601 | Neurocalcin-delta OS=Homo sapiens OX=9606 GN=NCALD PE=1 SV=2 | NCALD | 1.9611 | 0.0166 |
| P16278 | Beta-galactosidase OS=Homo sapiens OX=9606 GN=GLB1 PE=1 SV=2 | GLB1 | 1.9504 | 0.0297 |
| Q7L5A8 | Fatty acid 2-hydroxylase OS=Homo sapiens OX=9606 GN=FA2H PE=1 SV=1 | FA2H | 1.914 | 0.0398 |
| Q93008 | Probable ubiquitin carboxyl-terminal hydrolase FAF-X OS=Homo sapiens OX=9606 GN=USP9X PE=1 SV=4 | USP9X | 1.91 | 0.0075 |
| P08236 | Beta-glucuronidase OS=Homo sapiens OX=9606 GN=GUSB PE=1 SV=2 | GUSB | 1.8988 | 0.0143 |
| Q9BTY2 | Plasma alpha-L-fucosidase OS=Homo sapiens OX=9606 GN=FUCA2 PE=1 SV=2 | FUCA2 | 1.8654 | 0.0329 |
| Q96S55 | ATPase WRNIP1 OS=Homo sapiens OX=9606 GN=WRNIP1 PE=1 SV=2 | WRNIP1 | 1.8414 | 0.0409 |
| P51808 | Dynein light chain Tctex-type 3 OS=Homo sapiens OX=9606 GN=DYNLT3 PE=1 SV=1 | DYNLT3 | 1.8148 | 0.0172 |
| Q9Y223 | Bifunctional UDP-N-acetylglucosamine 2-epimerase/N-acetylmannosamine kinase OS=Homo sapiens OX=9606 GN=GNE PE=1 SV=1 | GNE | 1.7915 | 0.0316 |
| O00442 | RNA 3'-terminal phosphate cyclase OS=Homo sapiens OX=9606 GN=RTCA PE=1 SV=1 | RTCA | 1.7775 | 0.0305 |
| Q8N0X4 | Citramalyl-CoA lyase, mitochondrial OS=Homo sapiens OX=9606 GN=CLYBL PE=1 SV=2 | CLYBL | 1.7693 | 0.0434 |
| Q8WXE0 | Caskin-2 OS=Homo sapiens OX=9606 GN=CASKIN2 PE=1 SV=2 | CASKIN2 | 1.7577 | 0.0337 |
| A6NED2 | RCC1 domain-containing protein 1 OS=Homo sapiens OX=9606 GN=RCCD1 PE=1 SV=1 | RCCD1 | 1.7304 | 0.0414 |
| Q9NPH2 | Inositol-3-phosphate synthase 1 OS=Homo sapiens OX=9606 GN=ISYNA1 PE=1 SV=1 | ISYNA1 | 1.7102 | 0.0205 |
| Q68EM7 | Rho GTPase-activating protein 17 OS=Homo sapiens OX=9606 GN=ARHGAP17 PE=1 SV=1 | ARHGAP17 | 1.6715 | 0.0100 |
| O14730 | Serine/threonine-protein kinase RIO3 OS=Homo sapiens OX=9606 GN=RIOK3 PE=1 SV=2 | RIOK3 | 1.6642 | 0.0058 |
| Q9H0U6 | 39S ribosomal protein L18, mitochondrial OS=Homo sapiens OX=9606 GN=MRPL18 PE=1 SV=1 | MRPL18 | 1.6515 | 0.0401 |
| P17655 | Calpain-2 catalytic subunit OS=Homo sapiens OX=9606 GN=CAPN2 PE=1 SV=6 | CAPN2 | 1.6461 | 0.0428 |
| Q8NCF5 | NFATC2-interacting protein OS=Homo sapiens OX=9606 GN=NFATC2IP PE=1 SV=1 | NFATC2IP | 1.6222 | 0.0167 |
| Q5HYK3 | 2-methoxy-6-polyprenyl-1,4-benzoquinol methylase, mitochondrial OS=Homo sapiens OX=9606 GN=COQ5 PE=1 SV=2 | COQ5 | 1.6193 | 0.0482 |
| Q99523 | Sortilin OS=Homo sapiens OX=9606 GN=SORT1 PE=1 SV=3 | SORT1 | 1.6055 | 0.0007 |
| Q15019 | Septin-2 OS=Homo sapiens OX=9606 GN=SEPTIN2 PE=1 SV=1 | SEPTIN2 | 1.5778 | 0.0253 |
| Q9NUT2 | Mitochondrial potassium channel ATP-binding subunit OS=Homo sapiens OX=9606 GN=ABCB8 PE=1 SV=3 | ABCB8 | 1.575 | 0.0330 |
| Q96RL7 | Vacuolar protein sorting-associated protein 13A OS=Homo sapiens OX=9606 GN=VPS13A PE=1 SV=2 | VPS13A | 1.5612 | 0.0076 |
| Q07617 | Sperm-associated antigen 1 OS=Homo sapiens OX=9606 GN=SPAG1 PE=1 SV=3 | SPAG1 | 1.5604 | 0.0195 |
| Q9Y6E0 | Serine/threonine-protein kinase 24 OS=Homo sapiens OX=9606 GN=STK24 PE=1 SV=1 | STK24 | 1.5586 | 0.0443 |
| P11766 | Alcohol dehydrogenase class-3 OS=Homo sapiens OX=9606 GN=ADH5 PE=1 SV=4 | ADH5 | 1.5433 | 0.0478 |
| Q9H488 | GDP-fucose protein O-fucosyltransferase 1 OS=Homo sapiens OX=9606 GN=POFUT1 PE=1 SV=1 | POFUT1 | 1.5141 | 0.0313 |
